# Supplementary material for: Non-cytolytic re-engineering of a viral vaccine vector enables durable effector-memory T cell immunity by reinforcing type I IFN induction
Source: Mol Ther Nucleic Acids. 2026 Feb 2;37(1):102852. doi: 10.1016/j.omtn.2026.102852 (PMC12925560; doi:10.1016/j.omtn.2026.102852)
Supplement: Document S2. Article plus supplemental information [file mmc2.pdf]

Molecular Therapy
Nucleic Acids
Original Article

Non-cytolytic re-engineering of a viral vaccine vector enables durable effector-memory T cell immunity by reinforcing type I IFN induction

Matias Ciancaglini,[1] Robin Avanthay,[2,7] Anna-Friederike Marx,[1] Tiago Abreu-Mota,[1] Davide Finozzi,[1] Jonas Fixemer,[1] Florian Geier,[3,6] Dominik Burri,[3,6] Ingrid Wagner,[4] Ilena Vincenti,[4] Mario Kreuzfeldt,[4,5] Doron Merkler,[4,5] Gert Zimmer,[2,7] and Daniel D. Pinschewer[1]

[1]Department of Biomedicine, Division of Experimental Virology, University of Basel, 4009 Basel, Switzerland; [2]Institute of Virology and Immunology, 3147 Mittelhäusern, Switzerland; [3]Department of Biomedicine, Bioinformatics Core Facility, University of Basel, 4031 Basel, Switzerland; [4]Department of Pathology and Immunology, University of Geneva, 1211 Geneva, Switzerland; [5]Division of Clinical Pathology, Geneva University Hospital, 1206 Geneva, Switzerland; [6]Swiss Institute of Bioinformatics, Basel, Switzerland; [7]Department of Infectious Diseases and Pathobiology, Vetsuisse Faculty, University of Bern, 3012 Bern, Switzerland

Replication-deficient viral vector systems hold promise for CD8 T cell-based vaccination, but the molecular mechanisms accounting for platform-specific differences in immunogenicity remain ill-defined. When comparing prototypic single-cycle vaccine delivery platforms, we found that lymphocytic choriomeningitis virus (rLCMV)-based vectors, which are non-cytolytic, elicited more durable and effector-memory-differentiated CD8 T cell responses than vectors based on cytolytic vesicular stomatitis virus (rVSV). Hence, we re-engineered rVSV to be non-cytolytic (rVSVMq). This vector induced more durable and effector-differentiated CD8 T cell memory than the parental rVSV, and it afforded superior protection against *Listeria* challenge. Improved CD8 T cell responses of non-cytolytic rVSVMq were driven by a reinforced type I interferon (IFN-I) response and its direct sensing by vaccination-induced CD8 T cells. Many vector cargo-specific CD8 T cells in the splenic marginal zone of rVSVMq- or rLCMV-vaccinated mice were in contact with vector cargo-expressing cells that co-expressed IFN-I. In contrast, rVSV-vectored cargo-expressing contacts of specific CD8 T cells were largely IFN I-negative. Thereby, vaccination with non-cytolytic viral vectors offered an opportunity for CD8 T cells to integrate peptide-MHC (major histocompatibility complex) and IFN-I signals during priming. These mechanistic insights should help to refine vaccines aimed at eliciting durable and protective effector-memory CD8 T cell immunity.

INTRODUCTION

CD8 T cells represent a key pillar of adaptive immunity and an important line of defense against intracellular bacteria, tumors, and most viral diseases.[1–7] The recent severe acute respiratory syndrome coronavirus 2 (SARS-CoV-2) pandemic resulted in the rapid clinical development of vaccines that were based on novel platforms and has provided additional insights into the role of CD8 T cells in infection and vaccination. CD8 T cells represent a critical component of immunity against severe COVID-19 disease[8] and are indispensable to achieve control of SARS-CoV-2 replication.[9] Moreover, vaccination-induced CD8 T cell responses are more durable than antibody titers[10] and they can protect against severe SARS-CoV-2 infection in the absence of neutralizing antibodies.[11–13] Equally importantly, rapidly evolving SARS-CoV-2 readily escapes antibody responses[14] but remains susceptible to recognition by vaccination-induced CD8 T cells, which typically cross-react to a wide range of viral variants.[13,15,16] Under certain conditions, CD8 T cell immunity may even prevent the transmission of a respiratory virus.[17]

The development of CD8 T cell-based vaccines has long represented a difficult task, which in recent years has been eased by the advent of new delivery technologies including a variety of viral vectors.[18–20] Replication-competent viral vector platforms can, however, raise safety concerns,[21,22] especially in the context of prophylactic vaccination. These considerations argue in favor of replication-deficient vectors, some of which can induce immune responses that in magnitude are comparable to their replication-competent counterparts.[23–25] Vast differences can, however, be observed between viral vector platforms in their ability to elicit long-lived protective CD8 T cell immunity,[19,26] and it remains insufficiently understood which molecular determinants of viral vectors account for this important feature. Viral targeting to and activation of antigen-presenting cells is widely seen as one important mechanism.[19,27–29] Moreover, the amount and duration of antigen supply is commonly thought of as a determinant of the potency of CD8 T cell responses,[26,30–33] and in case of replicating viral vaccine vector platforms antigen expression can be augmented by transient blockade of the type I interferon (IFN-I) receptor (IFNAR).[34] In contrast, IFN-I signals have commonly been

Received 24 November 2025; accepted 29 January 2026;
https://doi.org/10.1016/j.omtn.2026.102852.

Correspondence: Daniel D. Pinschewer, Department of Biomedicine, Division of Experimental Virology, University of Basel, 4009 Basel, Switzerland.
E-mail: daniel.pinschewer@unibas.ch

© 2026 The Author(s).
Published by Elsevier Inc. on behalf of The American Society of Gene and Cell Therapy.
This is an open access article under the CC BY license (http://creativecommons.org/licenses/by/4.0/).

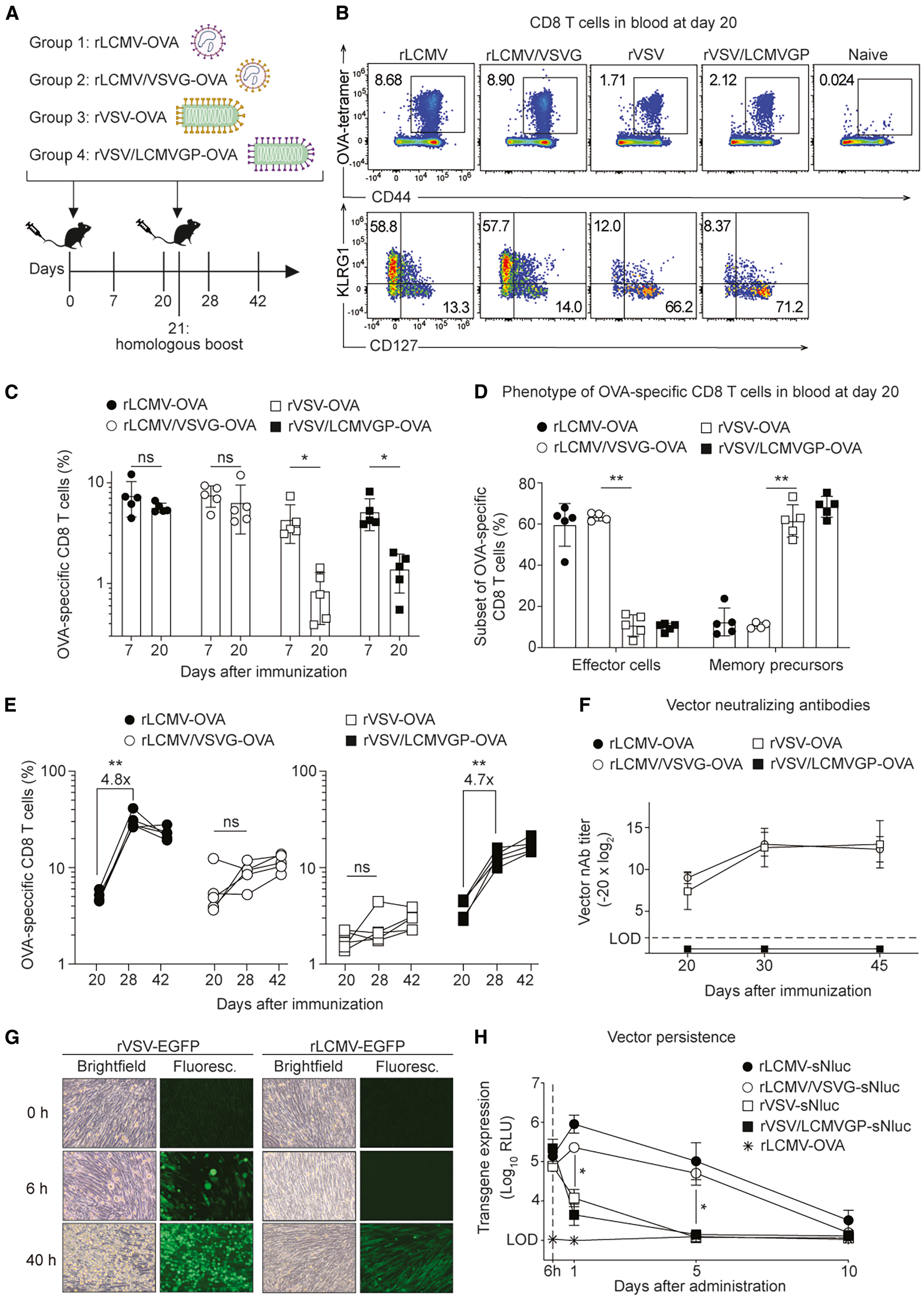

(legend on next page)

reported to improve or not to negatively impact the immunogenicity of replication-deficient platforms.[26,35,36] Yet viral vectors differ substantially in the levels of IFN-I they induce.[37–40] A further important characteristic of viral vectors to consider is their cytolytic or non-cytolytic behavior, which can impact the processing of vectorized cargo for presentation on major histocompatibility complex class I (MHC-I)[41] and viral interactions with the immune system more generally.[42] It remains, however, insufficiently understood how IFN-I induction and cytolytic behavior of replication-deficient viral vector platforms are interconnected and how these features translate into durable effector-memory CD8 T cells induction.

Vesicular stomatitis virus (VSV), the prototype member of the rhabdovirus family, has been studied by generations of immunologists for its ability to elicit potent and long-lived humoral immunity.[43–46] A VSV-based vaccine vector (rVSV/ZEBOV) expressing the Zaire Ebola virus (ZEBOV) glycoprotein has been tested in the 2014–2015 Ebola virus epidemic, demonstrating 100% prophylactic efficacy against Ebola virus disease,[47–49] and analogously engineered vaccines against Lassa fever are currently undergoing clinical development.[50] rVSV/ZEBOV induces T cell responses, too, but studies in non-human primates (NHPs) showed that protection against lethal challenge infection required vaccination-induced antibody immunity.[48,51] In stark contrast, an adenoviral vaccine expressing the same antigen protected NHPs by inducing CD8 T cell immunity,[4] suggesting that the two platforms differ in the arms of adaptive immunity they preferentially engage. One distinguishing feature of VSV and derived vector system consists in a prominent host cell shut-off, which is mediated by the viral matrix protein M that specifically blocks the nuclear export of cellular RNAs to the cytosol.[52–54] The host shut-off does not affect viral replication and transcription in the cytosol, ensuring high-level viral protein expression, but it results in rapid cytolytic cell death,[55,56] forming a basis for the utility of VSV and related rhabdoviruses in oncolytic cancer therapy.[57,58] Importantly, the M protein-mediated host shut-off is the only means how this virus suppresses the synthesis and release of IFN-I.[59] Whether and how a cytolytic life with host cell shut-off impacts CD8 T cell induction remains untested.

Here, we show that rVSV, when engineered to replicate in a non-cytolytic manner (rVSVMq), elicits more durable and effector-differentiated CD8 T cell memory with improved protective capacity. Superior immunogenicity of rVSVMq depended on its ability to elicit higher levels of systemic IFN-I, which signaled directly to antigen-specific CD8 T cells. Our observations provide a mechanistic link between non-cytolytic replication, systemic IFN-I responses, and potent effector-memory CD8 T cell immunity, which should help to rationally refine vaccination strategies.

RESULTS

rLCMV induces more durable effector-memory CD8 T cell responses than rVSV

To study and compare the ability of rVSV and lymphocytic choriomeningitis virus (rLCMV) vectors to induce CD8 T cell immunity, we employed replication-deficient vectors wherein the glycoprotein gene was replaced by the prototypic model antigen ovalbumin ([OVA]; rLCMV-OVA, rVSV-OVA). Both vectors are genetic vaccine delivery vehicles, requiring intracellular expression of the vector-encoded OVA to elicit immune responses. Accordingly, the production of these glycoprotein-deficient vectors necessitates the use of glycoprotein *trans*-complementing cell lines, which pseudotype budding particles to become single-round infectious.[19,60] To determine how the choice of viral surface glycoprotein impacted CD8 T cell induction, e.g., by influencing cell tropism, we generated not only rVSV-OVA and rLCMV-OVA carrying their respective own glycoprotein but also produced rLCMV vectors pseudotyped with VSV glycoprotein (rLCMV/VSVG-OVA) as well as rVSV vectors pseudotyped with LCMV glycoprotein (rVSV/LCMVGP-OVA), all of them single-round infectious. Each one of the four vectors was administered intravenously (i.v.) to mice, and OVA-specific CD8 T cell frequencies in blood were measured over time using MHC class I tetramers (Figures 1A–1D and S1A). At day 7 after immunization, all vectors elicited similar OVA-specific CD8 T cells (Figure 1C). By day 20 after prime, however, the responses elicited by rLCMV-OVA and rLCMV/VSVG-OVA remained stable, whereas rVSV-OVA- and rVSV/LCMVGP-OVA-induced CD8 T cells contracted 5- and 4-fold, respectively. These findings suggested that rLCMV-induced CD8 T cell responses were more durable than those induced by rVSV, irrespective of the glycoprotein used to pseudotype the vectors. Not only the durability but also the phenotype of responding CD8 T cells varied greatly between rLCMV- and rVSV-immunized mice. By day 20 after immunization the response to the former consisted predominantly in effector-memory CD8 T cells ($KLRG1^{+}$ $CD127^{-}$), while the response to the latter was dominated by more resting memory phenotype CD8 T cells ($KLRG1^{-}$ $CD127^{+}$) (Figures 1B and 1D).

Figure 1. rLCMV induces more durable effector-memory CD8 T cell responses than rVSV

(A) Experimental design. Mice were immunized intravenously with OVA-expressing rLCMV, rVSV, or their reciprocal pseudotypes on d0 and d21, and blood as well as serum samples were collected over time. (B) Representative FACS plots of OVA-tetramer-binding CD8 T cells and their phenotype in blood at d20. (C) Frequencies of OVA-tetramer-binding CD8 T cells in blood at d7 and d20. (D) Frequencies of OVA-tetramer-binding CD8 T cells with an effector ($KLRG1^{+}CD127^{-}$) or memory precursor ($KLRG1^{-}CD127^{+}$) phenotype in blood at d20. (E) Frequencies of OVA-tetramer-binding CD8 T cells in blood after primary immunization (d20) and after boost (d28, d42) with rLCMV- or rVSV-based vectors. (F) Vector-neutralizing antibodies in serum of mice vaccinated with rLCMV- or rVSV-based vectors over time. (G) Cytopathic effect of EGFP-expressing rLCMV and rVSV after infection of BHK21 cells. (H) Luciferase activity in serum of WT mice immunized with sNluc-expressing vectors pseudotyped with VSVG or LCMVGP. Samples from mice immunized with rLCMV-OVA were used to determine technical backgrounds. Symbols in (C, D, and E) represent individual mice with bars in (C and D) showing the mean ± SEM. Symbols in (F) show the mean ± SEM of $n = 5$ (F) or $n = 3$ (H) mice. One representative experiment of two similar ones is shown. Statistical analysis was performed by two-way ANOVA with Bonferroni's post-test for multiple comparisons (C, D, E, and H); ns: not significant; $^{*}p < 0.05$, $^{**}p < 0.01$, $p > 0.05$ was considered not statistically significant and is not indicated.

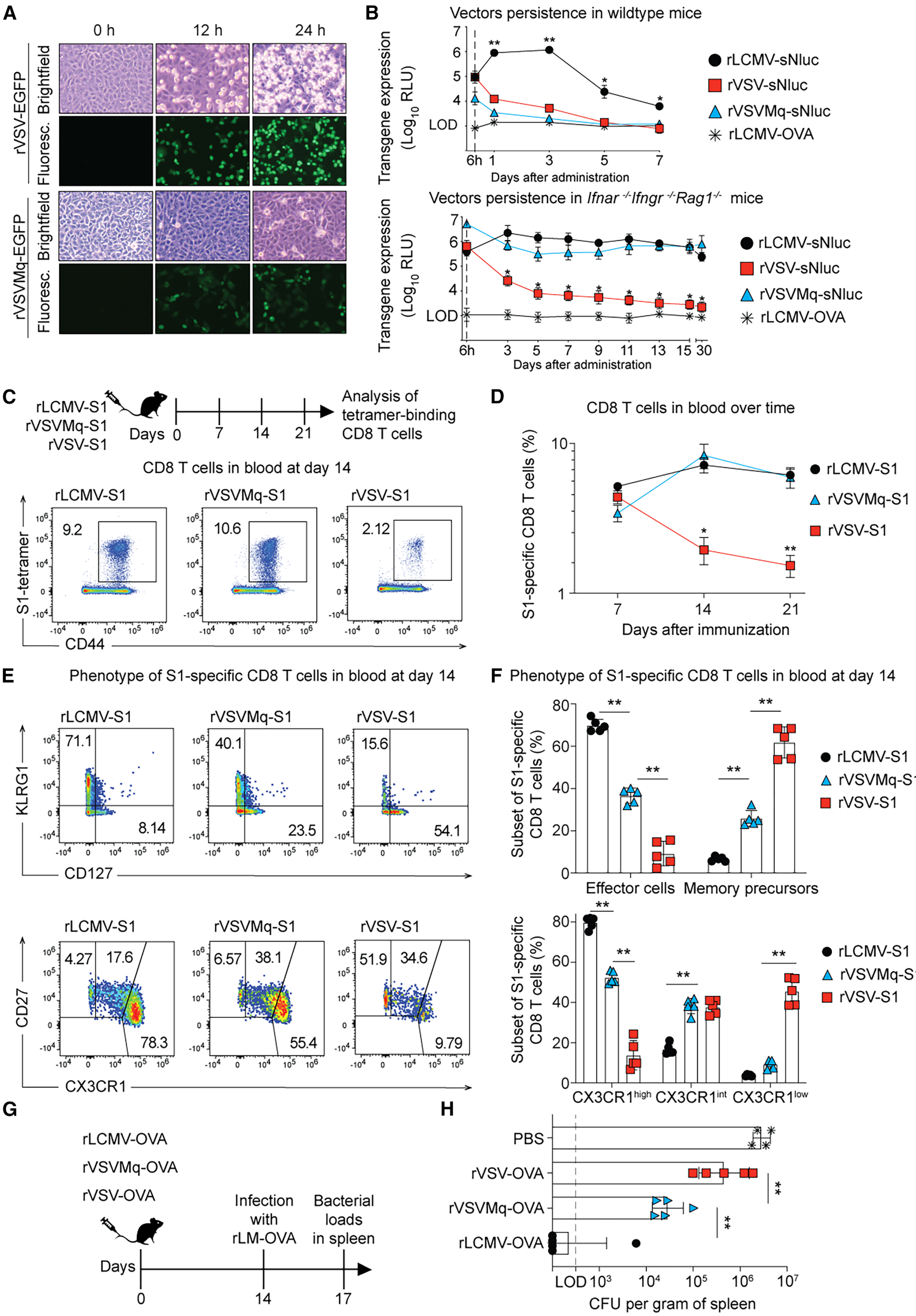

(legend on next page)

To test the ability of the various vector formats to boost OVA-specific CD8 T cell response upon re-administration, we performed a second immunization at day 21, in a homologous prime-boost regimen (Figure 1E). Booster vaccination using vectors pseudotyped with LCMV glycoprotein induced a significant increase in circulating OVA-specific CD8 T cells. By day 28 (7 days after boost), the frequencies of rLCMV-OVA- and rVSV/LCMVGP-OVA-induced CD8 T cells were augmented 4.8- and 4.7-fold, respectively. VSVG-pseudotyped vectors showed a trend toward higher CD8 T cell frequencies after booster vaccination, too, but these differences failed to reach statistical significance. The responses to all four vectors remained stable up to day 42 (21 days after boost). These results showed that even though the viral glycoproteins did not measurably influence the magnitude of vector-induced primary responses, they had a major impact on the secondary response upon homologous boosting, with vectors carrying LCMV-GP allowing for a more efficient boost of antigen-specific CD8 T cell response than VSVG-pseudotyped ones.

Pre-existing anti-vector immunity and notably vector-neutralizing antibodies can interfere with homologous prime-boost immunization regimens,[61] and the glycoproteins of LCMV and VSV differ substantially in their ability to elicit neutralizing antibodies.[46] To determine vector-neutralizing antibody induction by the different vectors, serum samples from immunized mice were assessed for their neutralization activity (Figure 1F). The sera of mice vaccinated with either one of the two vectors that carried LCMV glycoproteins, i.e., rLCMV-OVA and rVSV/LCMVGP-OVA, were devoid of detectable LCMV-neutralizing antibody activity, both prior to and after homologous boosting. On the contrary, VSV-neutralizing antibodies were readily induced after single immunization with VSV glycoprotein-pseudotyped rVSV-OVA and rLCMV/VSVG-OVA, and these neutralizing titers increased further after homologous boost. These data suggested that the differential ability of rVSV and rLCMV vectors to boost CD8 T cell responses were associated with the differential induction of vector-neutralizing antibodies after primary immunization.

Besides differential glycoprotein-mediated cell tropism, an important distinction between VSV and LCMV consists in their cytolytic and non-cytolytic life cycles, respectively. Unlike rLCMV, which replicates without any noticeable cytopathic effect, rVSV causes infected cells to round up and detach within 6 h after infection (Figure 1G). Taking into account that these replication-deficient vectors cannot propagate from cell to cell, antigen expression is limited to the first round of infected cells. Henceforth, the cytolytic activity of the vector can represent a limiting factor for the amount of antigen expressed *in vivo*, which in return is thought of as a major determinant of vector immunogenicity.[26,34,62] In order to quantify vectored antigen expression over time in mice, we generated vectors expressing secreted Nano-luciferase (sNluc), which can be sampled from serum to serve as a measure of the total amount of antigen expressed in the animal's body. i.v. administration of recombinant sNluc protein revealed that the protein's *in vivo* half-life ranged below 1 h, validating serum sNluc activity as a real-time surrogate of protein synthesis by vector-infected cells (Figure S1B). sNluc-expressing rVSV and rLCMV vectors were pseudotyped with either VSVG or LCMV-GP and were administered to mice i.v. (Figure 1H). Similar levels of sNluc activity were detected at 6 h after administration, irrespective of the vector used. By 24 h after administration, however, sNluc activity in animals receiving rVSV-based vectors had markedly declined, while sNluc expression by rLCMV-based vectors was still on the rise, largely independently of the glycoprotein used for pseudotyping. By day 5, rVSV-expressed sNluc had dropped below detection limits, while rLCMV-driven antigen expression subsided by around day 10. These results indicated that rLCMV can better persist in infected cells *in vivo,* expressing antigen for longer periods of time than rVSV. These observations raised the possibility that increased antigen availability for longer periods of time could have provided a better stimulation to CD8 T cells, culminating in the more durable and effector-differentiated CD8 T cell response observed.

Non-cytolytic rVSVMq induces long-lived effector-differentiated CD8 T cell memory and provides better protection against *Listeria* challenge than cytolytic rVSV

We hypothesized that abrogating rVSV's cytolytic activity might improve the CD8 T cell response. For this, we employed an attenuated VSV matrix protein quadruple mutant vector (rVSVMq), which lacks host shut-off activity and does not cause cytopathic effects in cell culture.[63] In keeping with earlier reports, rVSVMq cytolytic activity was abrogated, whereas rVSV infection induced a pronounced rounding and detachment of Vero E6 cells from the culture flask (Figure 2A). To test whether its non-cytolytic replication cycle prolonged rVSVMq antigen expression in mice, we turned again to

Figure 2. Non-cytolytic rVSVMq induces long-lived effector-differentiated CD8 T cell memory and provides better protection against *Listeria* challenge than cytolytic rVSV

(A) Cytopathic effect of rVSV-EGFP and rVSVMq-EGFP vectors on Vero E6 cells at 0, 12, or 24 h after infection. (B) Luciferase activity in serum of WT (top) and $Ifnar^{-/-}Ifngr^{-/-}Rag^{-/-}$ mice (bottom) immunized with sNluc-expressing vectors. Samples from mice immunized with rLCMV-OVA were used to determine technical backgrounds. (C) We immunized WT mice with rLCMV-S1, rVSVMq-S1, or rVSV-S1 on d0 and collected blood over time to analyze S1-tetramer-binding CD8 T cells. Representative FACS plots of S1-tetramer-binding CD8 T cells in blood at d14. (D) Frequencies of S1-tetramer-binding CD8 T cells in blood over time. (E) Gating strategy to identify S1-epitope-specific CD8 T cell subsets with an effector or memory precursor phenotype as judged based on KLRG1/CD127 (top) or CD27/CX3CR1 expression (bottom) at d14. (F) Frequencies of effector ($KLRG1^{+}CD127^{-}$) and memory precursor ($KLRG1^{-}CD127^{+}$) subsets (top) among S1-tetramer-binding CD8 T cells, and the abundance of subsets based on CX3CR1 expression levels in conjunction with CD27 (bottom) at d14 after immunization. (G) We immunized WT mice with rLCMV-OVA, rVSVMq-OVA, or rVSV-OVA and 14 days later, challenged them with OVA-expressing *Listeria monocytogenes* and collected spleens on d17. (H) Bacterial loads on d17 in spleen. Symbols in (B and D) represent the mean ± SEM of $n = 3$ (B) and $n = 5$ (D) mice. Symbols in (F and H) represent individual mice with bars indicating the mean ± SEM. One representative experiment of two similar ones is shown. Statistical analyses were performed by two-way ANOVA with Bonferroni's post-test for multiple comparisons (B, D, and F) or one-way ANOVA with Tukey's post-test (H) $^{*}p < 0.05$, $^{**}p < 0.01$, $p > 0.05$ was considered not statistically significant and is not indicated.

sNluc-expressing constructs (Figure 2B). By 6 h after administration to wild-type mice, rLCMV-sNluc and rVSV-sNluc expressed similar sNluc levels, whereas those of rVSVMq-sNluc-inoculated mice were about 10 times lower (Figure 2B, top). In keeping with the results of Figure 1H, rLCMV-vectored sNluc levels persisted at more or less constant levels up to day 3 after inoculation and remained over technical backgrounds for more than 7 days, whereas rVSV-vectored sNluc declined rapidly, reaching technical backgrounds by day 5. Despite the vector's non-cytolytic behavior, rVSVMq-expressed sNluc activity declined in parallel to the one produced by rVSV, reaching background levels already on day 3. The low and transient sNluc expression by non-cytolytic rVSVMq in wild-type mice suggested, therefore, that vector-extrinsic mechanisms influenced its persistence in the infected cell. For instance, interferon responses as well as adaptive immunity can have a major impact on antigen expression levels of viral vectors.[34,61,64] To test the intrinsic ability of the vectors to persist *in vivo*, i.e., their persistence in the absence of innate and adaptive host immune responses, we employed mice that lack type I and II interferon receptors as well as T and B lymphocytes (Figure 2B, bottom). Both non-cytolytic vectors, rLCMV-sNluc and rVSVMq-sNluc, maintained high levels of sNluc expression in these immunodeficient mice for more than 30 days, indicating that these single-round vectors persisted in the cells they had infected. In contrast, antigen levels expressed from the cytolytic rVSV-sNluc showed a steady decline, reaching background levels by around day 15. Given that rVSV and rVSVMq carried the same glycoprotein and hence targeted the same cell types, these data indicated that declining antigen levels observed for rVSV but not for rVSVMq were related to an intrinsic property of the respective vectors rather to the type of cells they transduced. Taken together, these findings suggested that in animals lacking IFN sensing as well as B and T cells, a non-cytolytic life cycle allowed the vector's persistence at fairly constant levels for prolonged periods of time, whereas cytolytic activity caused progressive loss of antigen expression, which was presumably reflective of host cell death.

Next, we set out to corroborate and extend our observations on differential OVA-specific CD8 T cell induction by rVSV and rLCMV (compare Figures 1A–1D) using a different antigen and to assess the impact of non-cytolytic replication on vector immunogenicity. rLCMV, rVSVMq, and rVSV vectors expressing the subunit 1 of the SARS-CoV-2 spike protein (S1) were administered to WT mice, and epitope-specific CD8 T cells in blood were analyzed over time using MHC class I tetramers (Figure 2C and 2D). Analogously to the observations made with OVA-expressing vectors, the responses elicited by rLCMV-S1 were more durable than those in response to rVSV-S1, which exhibited a more pronounced contraction by day 14 after administration (Figure 2D; compare Figure 1C). In remarkable contrast, immunization with rVSVMq-S1 induced frequencies and kinetics of S1 epitope-specific CD8 T cell that were similar to rLCMV-S1. Unlike rVSV-S1-induced CD8 T cell frequencies, which decreased by 2 weeks after immunization, the rVSVMq response expanded from day 7 to day 14 and was maintained at high frequencies until day 21. Similar observations were made in an independent experiment comparing analogous vectors expressing OVA (Figures S2A and S2B). Furthermore, we observed analogous kinetics when determining the total numbers of S1-specific CD8 T cells in spleens of mice immunized with the aforementioned vectors (Figure S2C and S2D). Another major difference between rLCMV- and rVSV-induced CD8 T cell responses was the phenotype (compare Figure 1D). rLCMV-S1-induced CD8 T cell responses were dominated by $KLRG1^{+}CD127^{-}$ and $CX3CR1^{+}CD27^{-}$ effector cells, whereas the rVSV-S1 response was biased toward the $KLRG1^{-}CD127^{+}$ and $CX3CR1^{-}CD27^{+}$ memory subsets (Figures 2E and 2F). In comparison to the latter cytolytic vector, non-cytolytic rVSVMq-S1 induced higher frequencies of both effector subsets. Albeit not reaching the levels obtained after rLCMV immunization, the $KLRG1^{+}CD127^{-}$ and $CX3CR1^{+}CD27^{-}$ populations were 5- and 4-fold higher than those induced by rVSV-S1, respectively. Similarly, the $KLRG1^{-}$ $CD127^{+}$ and $CX3CR1^{-}$ $CD27^{+}$ memory subsets elicited by rVSVMq-S1 made up for 25% and 10% of the epitope-specific CD8 T cell response, respectively, about midways between rLCMV and rVSV. S1-binding antibody responses on day 7 after rVSV-S1 immunization were significantly higher than those in response to rVSVMq-S1 and a similar trend was noted at later time points (Figure S2E), indicating that the rVSVMq vector design improved T cell but not B cell immunogenicity.

Finally, we compared the different vaccine vectors in a well-characterized model of CD8 T cell-mediated protection.[65] Mice were immunized with either one of the OVA-expressing vectors and challenged 2 weeks later with OVA-expressing *Listeria monocytogenes* (rLM-OVA), an intracellular bacterium that is controlled by CD8 T cells. Spleens were collected 3 days after infection to determine bacterial loads (Figure 2G and 2H). When compared to naive animals, all three vectors conferred some level of protection. Importantly, rVSV-OVA was only modestly protective, while rVSVMq-OVA-induced immunity suppressed bacterial loads to 100-fold lower levels than rVSV-OVA vaccination. Mice immunized with rLCMV-OVA were almost free of rLM-OVA, which may have been due, at least in part, to the particularly pronounced effector CD8 T cell profile[66] elicited by this vaccine (compare Figures 2E and 2F). Taken together, these data showed that the non-cytolytic rVSVMq-OVA induced more durable and more effector-differentiated CD8 T cell memory than rVSV-OVA, which correlated with substantially improved protection against *Listeria* challenge.

rVSVMq-induced CD8 T cells exhibit transcriptomic signatures of effector differentiation and IFN-I signaling

The kinetics and phenotype of rVSVMq-induced CD8 T cell responses were dissimilar to those elicited by rVSV but resembled the responses observed in rLCMV-immunized mice, prompting us to characterize them at the transcriptional level. We vaccinated animals with rVSV-S1, rVSVMq-S1, or rLCMV-S1; sorted tetramer-binding cells 7 and 14 days later; and processed them for single-cell RNA sequencing ([scRNA-seq], Figures 3A and S3A). Projection of the sequencing data onto a t-distributed stochastic neighbor embedding (t-SNE) dimensional reduction space

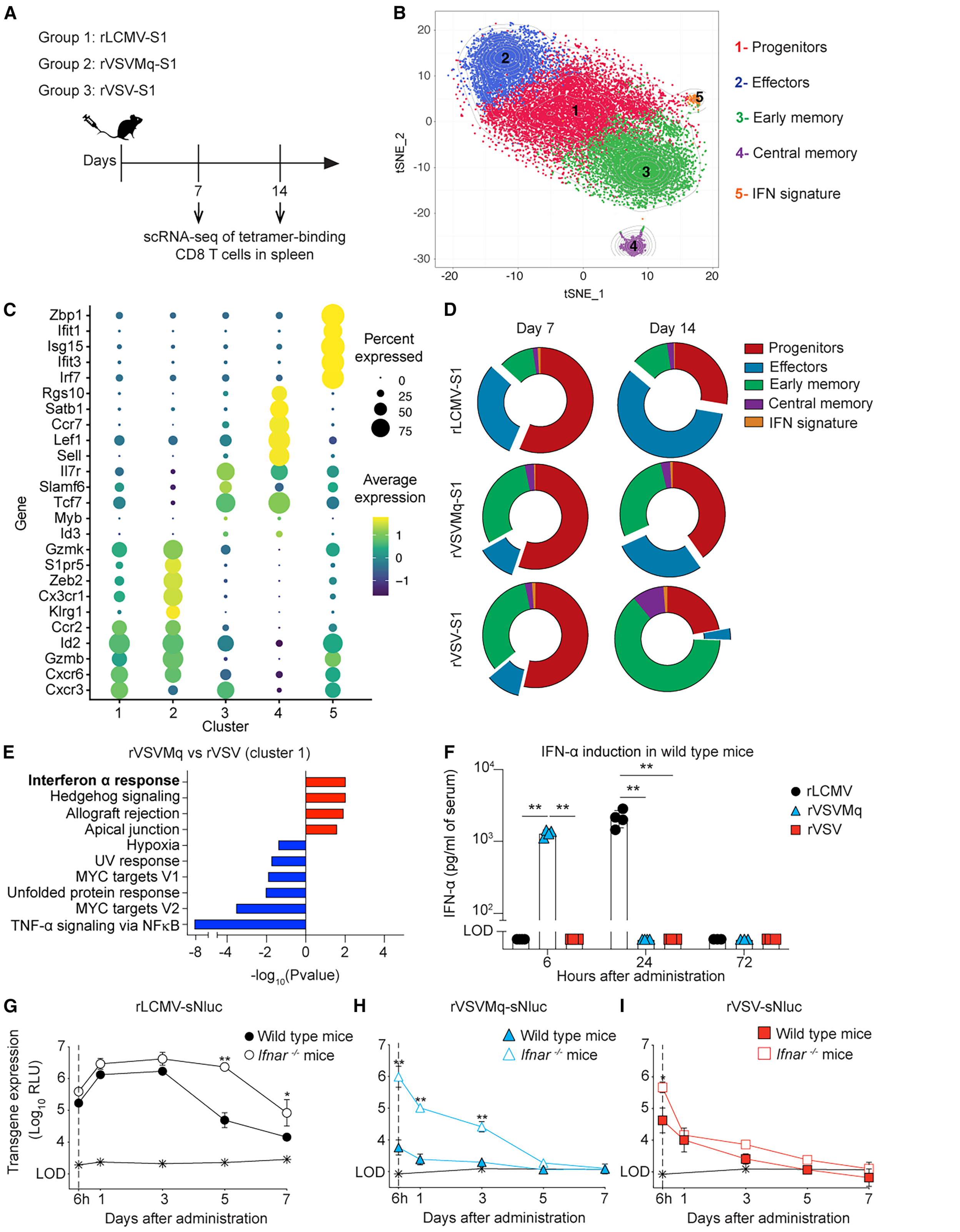

(legend on next page)

differentiated five clusters of cells (Figure 3B). The progenitor cluster 1 displayed moderate expression of the memory-associated transcription factor *Tcf7*, the survival cytokine receptor *Il7r*, and the progenitor marker *Slamf6*, in conjunction with intermediate expression of the chemokine receptor *Cxcr3* and moderate expression of effector-related genes such as *Gzmk* (Figure 3C). The effector cluster 2 contained copious amounts of transcripts for *Gzmb* (cytotoxic activity), the effector-associated surface markers *Cx3cr1* and *Klrg1*, sphingosine-1-phosphate receptor 5 (*S1pr5*),[67] and the effector differentiation-associated gene *Zeb2*.[68] Early memory cluster 3 cells were rich in transcripts for *Tcf7*, the anti-apoptotic regulator *Bcl2*, and the survival receptor *Il7r*. The central memory cluster 4 resembled cluster 3 but exhibited an increased expression of *Sell* (encoding for L-selectin) and CCR7. The interferon signature cluster 5 was enriched for IFN-stimulated gene transcripts such as *Irf7*.

Based on the relative abundance of these T cell clusters we compared the dynamics of CD8 T cell differentiation in rVSV-S1-, rVSVMq-S1-, and rLCMV-S1-vaccinated mice (Figures 3D and S3B). At day 7, all three vectors induced predominantly progenitor cells (cluster 1). T cell populations in the two rVSV-S1-vaccinated groups were similarly distributed, whereas rLCMV-S1-vaccinated animals showed some enrichment in effector cells (cluster 2) and a concomitant reduction in early memory cells (cluster 3). By day 14, the abundance of progenitor cluster 1 cells had proportionally declined in all groups. Of note, however, S1-specific CD8 T cells in rVSV-S1-vaccinated mice were shifted toward the early memory cluster 3, while rVSVMq-S1- and rLCMV-S1-induced responses were enriched in effector-differentiated cluster 2 cells. These transcriptional data corroborated our flow cytometric analyses and documented that rVSVMq-S1-induced CD8 T cell responses were more effector differentiated, resembling those elicited by rLCMV-S1.

To further compare CD8 T cell responses induced by rVSV-S1 and rVSVMq-S1 and to identify biological pathways enriched in rVSVMq-S1-induced CD8 T cells, we performed a gene set enrichment analysis (GSEA). GSEA revealed that rVSVMq-stimulated CD8 T cells from progenitor cluster 1 exhibited significantly higher expression of IFN-α-induced genes (Figure 3E), suggestive for more pronounced IFN-I signaling upon vaccination with the non-cytolytic rVSVMq-S1 vector than with cytolytic rVSV-S1. To directly test this hypothesis, we assessed systemic IFN-α levels in serum samples collected in the first 3 days after vector administration. Indeed, rVSVMq-S1 triggered a substantial systemic IFN-α response detectable at 6 hours after administration, whereas serum IFN-α of rVSV-S1-vaccinated mice remained below detection limits throughout the observation period (Figures 3F and S3C). In keeping with our earlier report,[69] rLCMV-S1 administration triggered a systemic IFN-α response peaking at 24 h after immunization.

Higher IFN-I responses to rVSVMq than to rVSV in conjunction with the known role of the VSV M protein as IFN-I antagonist[70,71] offered a compelling explanation for lower antigen expression levels by rVSVMq than by rVSV (compare Figure 2B). Hence, we analyzed vectored sNluc expression in IFN-I receptor-deficient (*Ifnar*$^{-/-}$) mice (Figures 3G–3I). Both rVSV-sNluc and rVSVMq-sNluc expressed more serum sNluc when administered to *Ifnar*$^{-/-}$ mice instead of WT controls. This difference was, however, much more pronounced for rVSVMq-sNluc than for rVSV-sNluc. At 6 hours after administration, rVSVMq-sNluc-vaccinated *Ifnar*$^{-/-}$ mice expressed 100 times more sNluc than WT controls (Figure 3H). These differences persisted over time, and by consequence, serum sNluc activity in rVSVMq-vaccinated *Ifnar*$^{-/-}$ mice was still over detection limits on day 3 after administration but had reached technical background levels in WT controls. In contrast, rVSV-vectored sNluc in the serum of *Ifnar*$^{-/-}$ mice was only ∼10-fold higher than in WT mice at 6 hours after administration, and no statistically significant differences were recorded at later time points (Figure 3I). rLCMV-vectored sNluc expression levels in *Ifnar*$^{-/-}$ and WT mice were not significantly different in the first 3 days and persisted for at least 7 days in both types of mice, which was supposedly due to the viral nucleoprotein's ability to dampen IFN-I signaling[72,73] (Figure 3G). Collectively, these findings established a correlation between the non-cytolytic replication cycles of rVSVMq and LCMV, the induction of systemic IFN-α responses by these vectors, and the sustained effector differentiation of CD8 T cells triggered by the same.

rVSVMq promotes IFNAR-dependent expansion of antigen-specific CD8 T cells

Virus-induced expansion of CD8 T cells can be promoted by IFN-I, the extent of which depends on the infecting pathogen,[74] prompting us to test whether the same applied to the vectors under study. We administered to mice an IFNAR-blocking antibody or isotype control followed by vaccination with rVSV-S1, rVSVMq-S1, or

Figure 3. rVSVMq-induced CD8 T cells exhibit transcriptomic signatures of effector differentiation and IFN-I signaling
(A) We immunize WT mice with rLCMV, rVSVMq, or rVSV expressing the S1 domain of the SARS-CoV-2 spike protein (Wuhan Hu-1 strain), and spleens were collected at d7 and d14. (B) Clustering of splenic S1 epitope-specific CD8 T cells from two mice per condition (vector used, time point), visualized using t-SNE. Each cell is represented by a point and colored by cluster. (C) Dotplot of gene expression levels of selected genes among clusters. (D) Differential abundance of CD8 T cells in each cluster, individually displayed for each vector and time point. (E) Gene set enrichment analysis of cluster 1 cells from day 14, comparing rVSVMq-induced CD8 T cells compared against those induced by rVSV. Gene set enrichment analysis was conducted and gene sets with an false discovery rate <0.01 are displayed. (F) IFN-α levels in serum of mice immunized with rLCMV-S1, rVSVMq-S1, or rVSV-S1. (G–I) Luciferase activity in serum of WT and *Ifnar* $^{-/-}$ mice immunized with sNluc-expressing rLCMV (G), rVSVMq (H), or rVSV (I). Symbols in (F) represent individual mice ($n = 4$ per group) with bars showing the mean ± SEM. Symbols in (G–I) show the mean ± SEM of 3 mice per group. One representative experiment of two similar ones is shown in (F, G, H, and I). Statistical analyses in (G–I) were performed by two-way ANOVA with Bonferroni's post-test for multiple comparisons, $^{*}p < 0.05$, $^{**}p < 0.01$, $p > 0.05$ was considered not statistically significant and is not indicated.

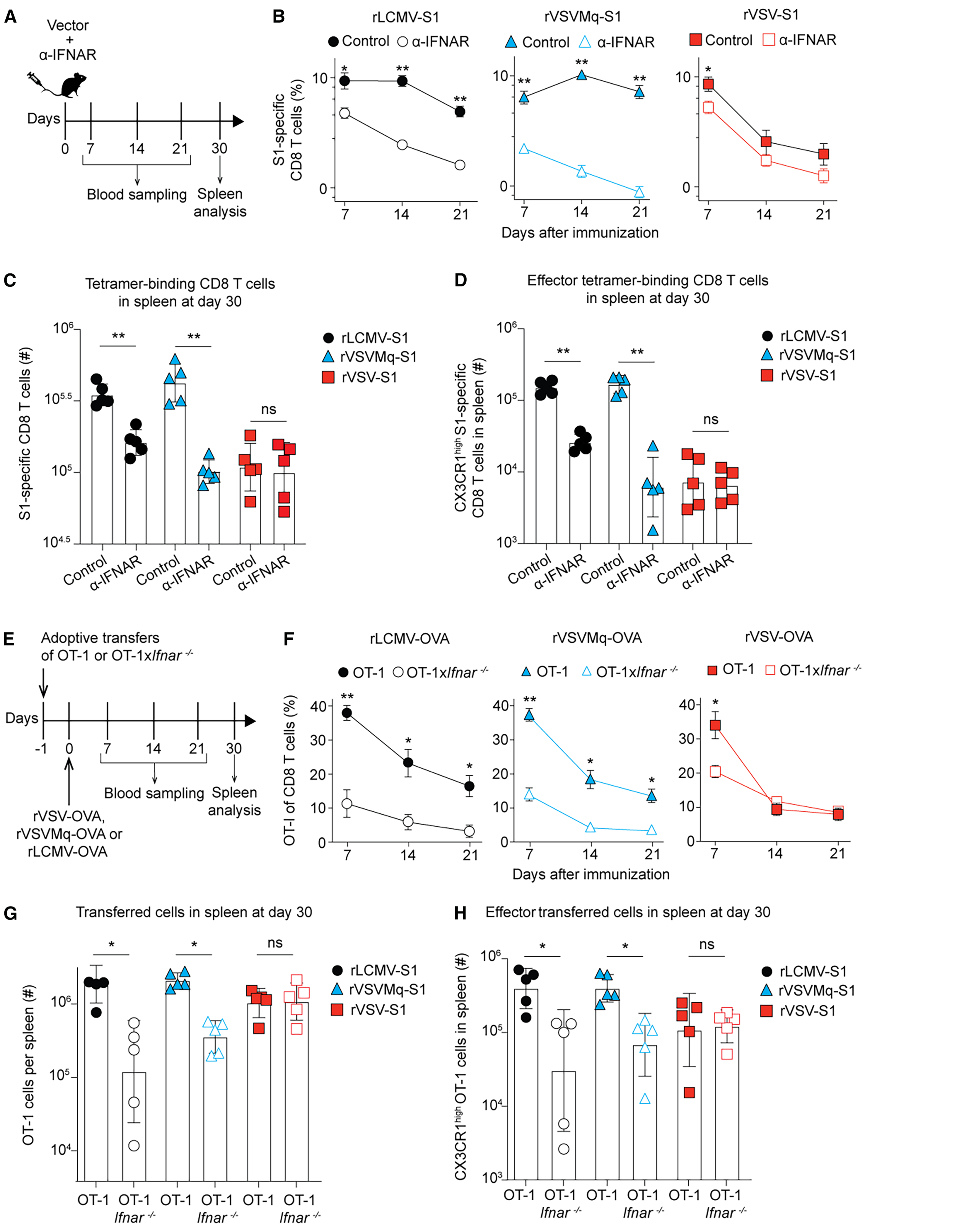

(legend on next page)

rLCMV-S1 expressing S1 and measured S1-specific CD8 T cells over time (Figure 4A). In rVSV-S1-vaccinated animals, IFNAR blockade reduced the frequency of circulating tetramer-binding CD8 T cells in blood 1.6-fold on day 7 but failed to exert significant effects at later time points (Figure 4B). In remarkable contrast, responses to rVSVMq-S1 and rLCMV-S1 were significantly suppressed at all time points when IFNAR was blocked. On day 30, the total number of S1-specific CD8 T cells in the spleen of rVSVMq-S1- and rLCMV-S1-vaccinated mice was 19.7-fold and 5.8-fold reduced, respectively, when IFN-I was blocked, whereas responses to rVSV-S1 were unaffected by the same (Figure 4C). IFNAR blockade exerted an at least equally pronounced effect on the number of CX3CR1$^+$CD27$^-$ effector CD8 T cells induced by rVSVMq-S1 and rLCMV-S1 but failed to have a detectable impact on the limited effector CD8 T cell response to rVSV-S1 (Figure 4D). These data showed that IFNAR signaling was a key determinant of both the magnitude and effector differentiation of CD8 T cell responses to rVSVMq-S1 and rLCMV-S1 but was largely dispensable for rVSV-S1-driven CD8 T cell induction.

To determine whether IFN-I-driven CD8 T cell expansion and differentiation depended on CD8 T cell-intrinsic IFNAR signaling, we adoptively transferred OVA-specific OT-1 CD8 T cells that were either *Ifnar*-deficient or -sufficient (OT-1, OT-1x*Ifnar*$^{-/-}$), followed by immunization with rVSV-OVA, rVSVMq-OVA, or rLCMV-OVA (Figures 4E and S4). Recipients were natural killer (NK) cell-depleted to avoid NK cell-mediated killing of *Ifnar*-deficient T cells.[75,76] On day 7 after rVSV-OVA immunization, *Ifnar*-sufficient OT-1 cells reached ~2-fold higher frequencies than OT-1x*Ifnar*$^{-/-}$ cells. This difference was, however, lost by day 14, and both cell populations persisted at comparable frequencies throughout day 21 (Figures 4F and S4). In contrast, when triggered by rVSVMq-OVA- or rLCMV-OVA, OT-1 cell frequencies in peripheral blood were significantly higher than those of OT-1x*Ifnar*$^{-/-}$ cells from day 7 onward throughout day 21. Cell counts in the spleen on day 30 paralleled these findings in blood (Figure 4G). OT-1 and OT-1-IFNAR$^{-/-}$ cell counts were comparable in rVSV-vaccinated animals, whereas in rVSVMq- and rLCMV-immunized animals, OT-1 cell numbers exceeded those of OT-1x*Ifnar*$^{-/-}$ cell by 5.4- and 8.9-fold, respectively. Analogous findings were made when enumerating CX3CR1$^+$CD27$^-$ effector memory progeny of OT-1 and OT-1x*Ifnar*$^{-/-}$ cells (Figure 4H). These results showed that more potent CD8 T cell expansion and effector differentiation in response to rVSVMq-OVA and rLCMV-OVA immunization depended to a significant extent on direct IFN-I signaling to antigen-specific CD8 T cells.

rVSVMq but not rVSV vaccination offers spatial integration of cognate antigen and IFN-I signals by antigen-specific CD8 T cells

The above experiments indicated that the IFN-I response elicited by rVSVMq was directly sensed by vector-induced CD8 T cells, significantly promoting their expansion and effector differentiation. To further dissect the spatial relationship between vector-transduced cells, IFN-I-producing cells, and antigen-specific CD8 T cells, we adoptively transferred OT-1 cells into wild-type mice, followed by OVA-expressing vector immunization. Spleens were analyzed 6, 12, 18, and 24 h later (Figures 5 and S5). IFN-α-expressing cells were most abundant at 6 hours after rVSV-OVA or rVSVMq-OVA immunization and at 24 h after rLCMV-OVA, corresponding to the latter two vectors' respective serum IFN-α peak (Figure S5; compare Figure 3F). Immunohistochemical analyses of tissue sections revealed that all three vectors targeted predominantly the marginal zone (Figures 5B–5D).

A substantial proportion of adoptively transferred OT-1 cells were localized in the marginal zone, some of them in immediate proximity to vector-infected cells (Figures 5B–5D), suggesting early cognate interactions. Importantly, in spleens of rVSVMq-OVA- and rLCMV-OVA-vaccinated animals, the marginal zone cells in contact with OT-1 cells often produced IFN-α (Figures 5B and 5C). OT-1 cells in the spleen of rVSV-vaccinated animals were in contact with vector-transduced cells, too, but the latter lacked detectable IFN-α production (Figure 5D). In the spleen of rVSVMq-OVA- and rLCMV-OVA-vaccinated mice, ~2%–3% of vector-infected cells produced IFN-α at levels detectable by histology, while such cells were very rare or absent in rVSV-OVA-immunized animals (Figure 5E). This differential abundance of IFN-α-producing cells in spleen paralleled serum IFN-α levels in the serum of mice (compare Figure 3F). Importantly also, OT-1 cells in rVSVMq-OVA- and rLCMV-OVA-immunized spleens were often found in contact with vector-infected cells producing IFN-α, while such contacts were virtually undetectable in rVSV-OVA-infected spleens (Figure 5F).

Taken together, these observations suggested that antigen-specific CD8 T cells engaged with vector-transduced cells in the marginal zone as early as 6 hours after rVSV-OVA and rVSVMq-OVA immunization and 24 h after rLCMV-OVA vaccination. Importantly,

Figure 4. rVSVMq promotes IFNAR-dependent expansion of antigen-specific CD8 T cells

(A) We treated mice with either anti-IFNAR antibody or isotype control and immunized them with rVSV-S1, rVSVMq-S1, or rLCMV-S1 on d0. We analyzed blood over time and collected the spleen on d30. (B) Frequencies of S1 epitope-specific CD8 T cells in blood over time. (C) Splenic count of total S1 epitope-specific CD8 T cells in spleen on d30. (D) Splenic count of S1 epitope-specific CX3CR1^{high} effector CD8 T cells in spleen on d30. (E) We transferred 2,000 OT-1 or OT-1x*Ifnar*$^{-/-}$ cells i.v. at d−1 and immunized the recipients at d0 with rVSV-OVA, rVSVMq-OVA or rLCMV-OVA. NK cell-depleting antibody was administered on d−1 and d1. (F) Frequencies of OT-1 and OT-1x*Ifnar*$^{-/-}$ cells in blood over time. (G) Total numbers of OT-1 and OT-1x*Ifnar*$^{-/-}$ cells in spleen at d30. (H) Splenic count of OT-1 and OT-1x*Ifnar*$^{-/-}$ cells with an effector phenotype (CX3CR1^{high}) at d30. Symbols in (C, D, G, and H) represent individual mice with bars showing the mean ± SEM. Symbols in (B and F) show the mean ± SEM of 5 mice per group. One representative experiment of two similar ones is shown. Statistical analyses were performed with two-way ANOVA with Bonferroni's post-test for multiple comparisons (B, C, D, F, G, and H); ns: not significant; *$p < 0.05$, **$p < 0.01$.

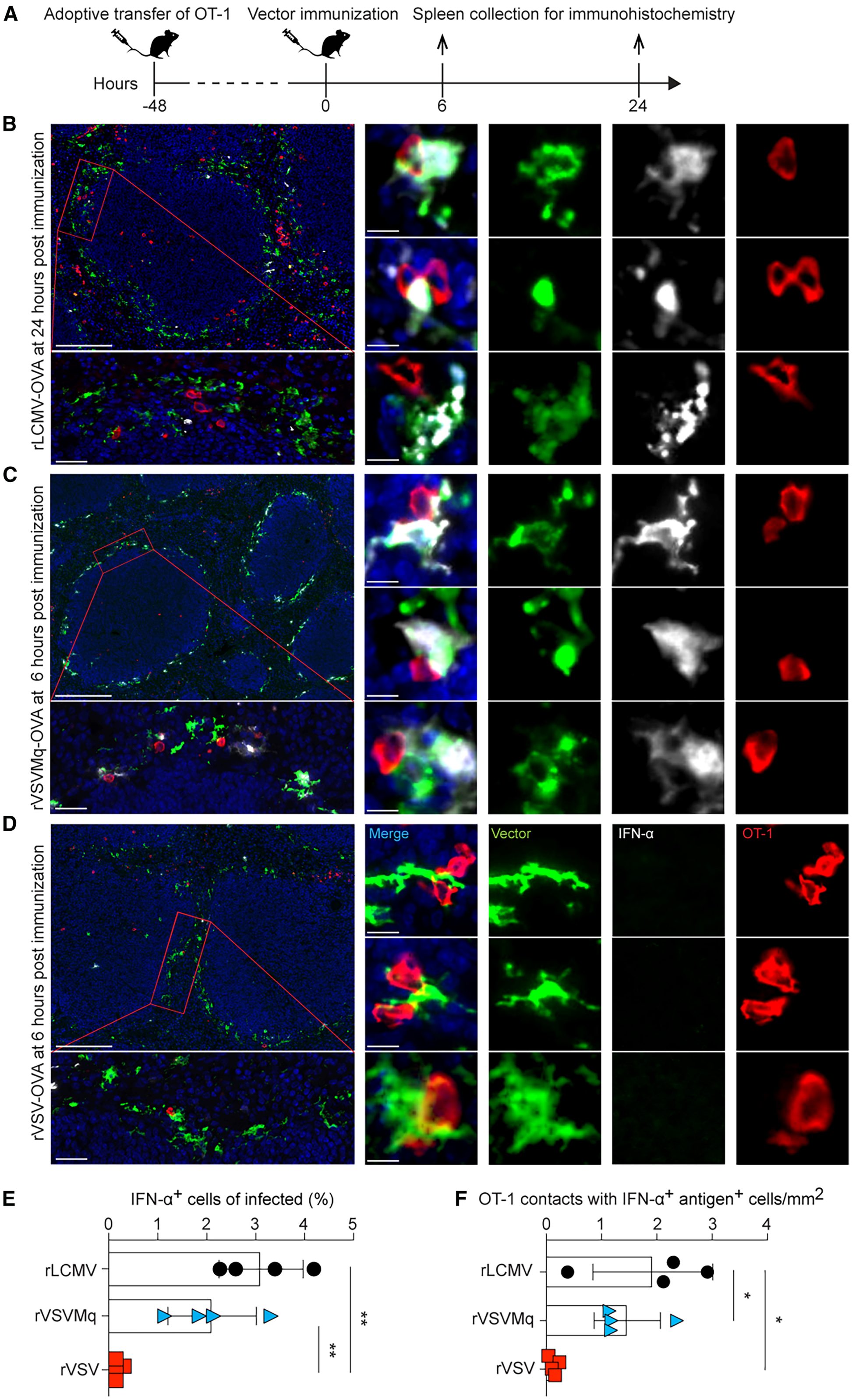

(legend on next page)

however, these rVSVMq-OVA- and rLCMV-OVA-infected cells not only expressed cognate antigen but produced also IFN-I, supposedly offering an opportunity for CD8 T cells to integrate T cell receptor and IFN-I receptor signaling.

DISCUSSION

The induction of robust and durable effector-memory CD8 T cell immunity holds great promise for both prophylactic and therapeutic vaccination against a range of infectious diseases and tumors. Replication-deficient viral vectors represent a preferred modality to induce such responses, and owing to their safety and reactogenicity profile, they are attractive platforms for clinical translation. Platform-specific differences in immunogenicity can, however, be substantial, and the biological features determining potent CD8 T cell immunity are mechanistically ill-defined. In this study we demonstrate that rendering a cytolytic vector non-cytolytic can substantially augment its ability to induce durable and protective effector-memory-differentiated CD8 T cell immunity. The pronounced effects of non-cytolytic vector replication were evident at the phenotypic and transcriptional levels of antigen-specific CD8 T cells and translated into substantially improved protection against *Listeria monocytogenes* challenge. Mechanistically, we show that non-cytolytic vector replication augments IFN-I production and that IFN-I sensing by CD8 T cells is key for durable effector-memory formation. These observations raise the possibility that IFN-I secretion by antigen-presenting cells in the priming phase of the CD8 T cell response enables the spatiotemporal integration of T cell receptor signals with IFN-I, often referred to as “signal 3” in T cell activation, thereby promoting CD8 T cell proliferation, survival, and effector differentiation.[77]

Earlier studies have shown that IFN-I released upon viral infection can act directly on CD8 T cells to support clonal expansion and memory formation and in the context of mRNA vaccination, IFN-I is essential for CD8 effector T cell differentiation.[78] While initial findings in LCMV-infected mice had suggested interferon signaling in CD8 T cells was virtually indispensable for these cells' antiviral response,[79] subsequent studies showed that much of this effect was due to NK cell-mediated killing of IFNAR-deficient CD8 T cells.[75,76] Accordingly, the relative impact of IFN-I on CD8 T cell responses to infection varied considerably between pathogens,[80–83] which may have been due, at least in part, to differential levels of NK cell activity elicited. Reports on the role of IFN-I in the context of vectored vaccination appeared inconsistent. IFN-I responses elicited by the replication-deficient modified vaccinia virus Ankara were required for optimal humoral and cellular immune responses,[35,36] whereas in the context of adenovirus-vectored vaccination, IFN-I was shown to limit CD8 T cell expansion.[26] Transient blockade of IFN-I signaling was proposed as a strategy to augment immune responses to various life-attenuated, replicating vaccine vectors, supposedly by increasing and/or extending antigen expression.[34] In addition, transient IFNAR blockade was shown to result in a delayed but overshooting IFN-I response that was associated with reinforced IFN-I- and costimulation-induced gene expression signatures in vaccination-elicited CD8 T cells.[34] These findings align closely with the observations and conclusions drawn in the present study. The notion that a vector's cytolytic or non-cytolytic replication cycle can profoundly alter the IFN-I dependence of the CD8 T cell response it elicits may help reconciling some of the seemingly contradictory reports on the impact of IFN-I on T cell responses to different vector platforms.[26,35,36] Higher systemic IFN-I responses in non-cytolytic infection, potentially due to continuous intracellular replication of non-cytolytic vectors and incessant activation of cytoplasmic RNA sensors such as RIG-I,[84] may simply result in a more pronounced effect on immune responses. Additionally, non-cytolytic infection and higher IFN-I responses may engender altered pathways of innate immune activation, cytokine production,[85] and dendritic cell maturation as reported for VSV-expressing non-cytolytic M protein variants.[70,86,87] Differential TNF-alpha signaling in rVSV- and VSVMq-induced T cells, as suggested by the GSEA analysis in Figure 3E, may be of relevance, too, and warrants future investigation.

Unlike for CD8 T cell immunity, antibody responses to rVSVMq-vectored cargo were somewhat lower or at best equivalent to those elicited by rVSV. Reduced levels of transgene expression by VSVMq (compare Figure 2B) as well as detrimental IFN-I effects on B cell responses[88–90] may have contributed to this outcome. As an additional observation of interest, glycoprotein swap between rVSV and rLCMV did not substantially affect primary CD8 T cell responses. Albeit a main determinant of receptor usage and tropism,[91–93] vectors pseudotyped with LCMV-GP or VSVG have been reported to infect dendritic cells with comparable efficiency,[85] which may explain the present findings. In stark contrast to prime vaccination, however, the choice of vector glycoprotein had a strong

Figure 5. rVSVMq vaccination enables co-localization of cognate antigen and IFN-I near antigen-specific CD8 T cells

(A) We transferred 2×10^6 OT-1 cells to mice at −48 h and immunized them with rVSV-EGFP-OVA, rVSVMq-EGFP-OVA, or rLCMV-OVA at 0 h. Spleens of rVSV-EGFP-OVA- (D) and rVSVMq-EGFP-OVA- (C) immunized mice were collected at 6 h and those of rLCMV-OVA-immunized mice at 24 h (B), corresponding to the peak of the serum interferon response in the respective immunization regimens (compare Figures S5 and 3F). (B) Spleen sections were stained for cell nucleus (blue), IFN-α (white), CD45.1 (red; OT-1 cells), and LCMV nucleoprotein (vector infection; green). (C and D) Spleen sections were stained for cell nucleus (blue), IFN-α (white), CD45.1 (red; OT-1 cells). and GFP (vector infection; green). (E) Percentage of IFN-α-expressing cells among virus antigen-positive cells on histological sections. (F) Number of OT-1 cell contacts with IFN-α-positive, virus antigen-positive cells per mm^2 of histological section. Scale bars: 100 μm (lowest magnification), 20 μm (intermediate magnification), and 5 μm (highest magnification). Representative areas of spleen tissue from whole spleen sections of 5 mice per group, originating from two independent experiments, are shown. Statistical analyses in (E and F) were performed with one-way ANOVA with Tukey's post-test; $^{**}p < 0.01$, $^{*}p < 0.05$, $p > 0.05$ was considered not statistically significant and is not indicated.

impact on the ability to augment CD8 T cell responses by homologous booster vaccination. The latter was associated with differential vector-neutralizing antibody induction by LCMV-GP- and VSVG-pseudotyped vectors, as previously reported.[46,94,95]

In summary, this study provides mechanistic insights into the ability of non-cytolytic replication-deficient viral vector systems to induce potent and durable effector-memory CD8 T cell immunity. This refines our understanding how vaccine vector-intrinsic properties are interlinked with adaptive immune responses and helps to rationally harness innate immune signaling for vaccination-induced cellular immunity against intracellular pathogens and tumors.

Limitations of the study

As evident from the literature,[26,34–36,79–83] the relative importance of IFN-I signals for CD8 T cell responses is context dependent. Henceforth, the relative importance of the present findings may vary somewhat between vector platforms. Moreover, cytokine signals other than IFN-I can exert profound context-dependent effects on CD8 T cell induction as exemplified by the proportionally higher impact of IL-12 on bacterially as opposed to virally induced CD8 T cell responses.[96,97] Accordingly, the extent to which individual vaccine platforms maintain the ability to promote CD8 T cell responses under IFNAR blockade may depend on each vector's capacity to activate alternative "signal 3" pathways. Last but not least, these findings in mice are yet to be confirmed in humans or non-human primates.

MATERIALS AND METHODS

Animal experiments

C57BL/6 wt mice were originally purchased from Charles River laboratories and bred locally under specific-pathogen-free conditions for colony maintenance and experiments. IFN-α/β-receptor- IFN-γ-receptor- and RAG1-triple-deficient mice (*Ifnar*$^{-/-}$*Ifngr*$^{-/-}$*Rag1*$^{-/-}$),[98] IFN-α/β-receptor-deficient mice (*Ifnar*$^{-/-}$),[99] and OT-1 mice,[100] all on a C57BL/6 background, have been described previously. *Ifnar*$^{-/-}$ mice and OT-1 mice were intercrossed to obtain OT-1x*Ifnar*$^{-/-}$ mice. Experimental groups were sex and age matched. Mice were bred at the ETH Phenomics Center Zurich (EPIC), whereas experiments were performed at the University of Basel in accordance with the Swiss law for animal protection and with permission from the Cantonal Veterinary Office of Basel City. Vector immunizations were performed at a dose of 1E+06 plaque-forming units (PFU) per mouse unless specified differently and administered in a volume of 200 μL into the tail vein.

Vector generation and titration

rLCMV-OVA has been described.[101] rLCMV-S1 and rLCMV-sNluc were designed and generated analogously, with open reading frames (ORFs) encoding the subunit 1 of SARS-CoV-2 spike protein (strain Wuhan-Hu-1, comprising the immunodominant epitope VNFNFNGL) and the secreted nanoluciferase (Promega), respectively, replacing the viral glycoprotein ORF.[101] Unless stated differently, the rLCMV vectors were grown in stably transfected LCMV-GP-expressing BHK-21 (referred to as BHK-23) cells[101] and were titrated by immunofocus assays[46] on stably LCMV-GP-expressing 293T cells.[101] For the generation of rVSV and rVSVMq vectors, the VSVG-deleted genomic plasmids pVSVΔG-EGFP and pVSVMqΔG-EGFP[63] were engineered to express ovalbumin, the subunit 1 of SARS-CoV-2 spike protein, or the secreted nanoluciferase genes from an additional viral transcription start-stop cassette. Unless stated differently, rVSV and rVSVMq vectors were grown on VSVG-*trans*-complementing BHK-G43 cells and titrated as previously described.[60]

In order to generate VSVG-pseudotyped rLCMV vectors, the latter were grown in BHK-G43 cells[63] to produce rLCMV/VSVG. In brief, expression of VSVG in BHK-G43 cells was induced by adding mifepristone at 10^{-9} M concentration to the culture medium for 6 h. After incubation, the cells were infected with rLCMV at MOI 0.01 for 3 h, the inoculum was washed away with PBS, and the cells were incubated with fresh media at 37°C. Vectors were harvested 72 h after infection and stored at −80°C. rVSV vectors were grown in BHK23 cells,[101] infected at an MOI = 0.1 for 24 h, to produce rVSV/LCMVGP.

Flow cytometry

Blood samples were collected and stained freshly with antibodies against CD45R/B220 (RA3-6B2), CD8 (53-6.7), CD44 (IM7), CD62L (MEL-14), CD127 (A7R34), Klrg1 (2F1), CX3CR1 (SA011F11), CD27 (LG3A10), and CD43 (1B11) purchased from BioLegend. Subsequently the stained samples were treated with fluorescence-activated cell sorting (FACS) lysing solution (BD Biosciences, Cat. #349202) to remove erythrocytes and fix the cells. For detection of S1-specific CD8 T cells, H2-K^{b} tetramers were conjugated to phycoerythrin (PE) and loaded with the SARS-CoV-2 spike-derived immunodominant peptide epitope VNFNFNGL. For detection of ovalbumin-specific CD8 T cells, H2-D^{b} tetramers were conjugated to PE and loaded with the SIINFEKL epitope. Peptide-MHC tetramers were prepared by the University of Lausanne tetramer core facility. The tetramers were added to the antibody mix for staining. Spleens were mechanically disrupted using a metal mesh and a syringe plunger, and cells were counted with an Immunospot S6 device (C.T.L.). For surface staining, splenocytes were incubated with the same cocktail of antibodies and tetramers as described above for blood, with the addition of anti-erythroid cell antibody (TER-119) as a dump gate. Dead cells were stained with Zombie-NIR Fixable Viability Kit (BioLegend, Cat: #423105). Samples were fixed by incubation with 2% paraformaldehyde for 15 min at room temperature. All samples were measured on a LSRFortessa flow cytometer (Becton Dickinson) or a 5-laser Aurora spectral flow cytometer (Cytek Biosciences, Fremnont, CA, USA) and analyzed with FlowJo software (BD Biosciences).

Virus neutralization assays, determination of serum luciferase activity, and ELISA assays for the quantification of S1-specific antibodies and IFN-α in serum

Microtainer tubes (Becton Dickinson) were used for serum collection. Immunofocus reduction assays were performed for the detection of LCMV-neutralizing antibodies.[102] VSV-neutralizing antibodies were measured by plaque reduction assays.[103]

Luciferase luminescence in serum samples was measured using the Nano-Glo Luciferase Assay Kit (Promega, Madison, USA), a Saphirell Tecan Infinite M-plex plate reader and white 96-well luciferase plates (Thermo Fisher Scientific Nunc A/S). For determination of the *in vivo* half-life of sNluc, HEK-293T cells were transfected with the mammalian expression vector pCAGGS expressing the sNluc gene using Lipofectamine 2000 (Thermo Fisher). After 24 h, cell culture supernatant was harvested to determine luciferase activity. Supernatant was administered i.v. into the tail vein of C57BL/6 mice, and blood samples were collected over time to determine Luciferase activity. The half-life of sNluc was determined by non-linear regression and one-phase decay parameters using GraphPad Prism v.10.2.1.

For detection of S1-specific antibodies, high-binding 96-well flat bottom plates (Sarstedt AG & Co.KG) were coated with 50 ng of spike protein subunit 1 of SARS-CoV-2 (GenScript) per well in 50 μL coating buffer overnight at 4°C. Plates were washed twice with PBS-T (0.05% Tween 20/PBS) and then blocked with 200 μL 5% BSA/PBS-T at room temperature for 45 min. In a separate plate, 2-fold serial dilutions of serum samples were performed in blocking solution, and the serially diluted sera were transferred to the spike protein-coated plates and incubated at 37°C for 1 h followed by five washes with PBS-T. Peroxidase-conjugated polyclonal anti-muse antibody (1:2,000 in blocking solution; Jackson, 115-035-062) was added, and the plates were incubated at 37°C for 60 min. After washing five times with PBS-T, HRP activity was detected using ABTS as a chromogen (Pierce) and the absorbance was measured at 405 nm using the Saphirell plate reader (Tecan). Arbitrary units are computed as ln(1,000 × A491nm); the limit of detection was determined as the maximum value reached with naive control serum.

Concentrations of IFN-α in mouse serum were determined using the VeriKine Mouse Interferon Alpha ELISA Kit according to the manufacturer's instructions (PBL Assay Science). This immunoassay is based on the international reference standard for mouse IFN-α, and it detects all 14 mouse IFN-α subtypes.

Listeria challenge

The recombinant *Listeria monocytogenes* expressing ovalbumin has been described.[104] The bacteria were grown in blood-heart infusion media (Sigma-Aldrich) at 37°C, harvested during the exponential growth phase and washed with phosphate-buffered saline. A dose of 10^3 colony forming units (CFU) was administered i.v. to mice.

scRNA-seq and bioinformatic analyses

Splenic single-cell suspensions were prepared as described for flow cytometric analysis. CD8 T cells were enriched by magnetic-activated cell sorting using the CD8+ T cells Isolation Kit, mouse (STEMCELL), but the antibody mix in the kit was replaced by a mix containing the following biotin-conjugated antibodies from BioLegend: anti-B220 (RA3-6B2), anti-CD19 (6D5), Ly-76 (TER-119), anti-CD4 (H129.19), and anti-CD138 (281-2). Cell suspensions were stained with antibodies against CD45R/B220 (RA3-6B2), CD8 (53-6.7), and DAPI purchased from BioLegend, PE-conjugated H2-K^b tetramers loaded with the SARS-CoV-2 spike epitope VNFNFNGL (produced by the University of Lausanne Tetramer core facility). Tetramer-binding CD8 T cells were FACS-sorted (FACS Aria II, BD), and the recovered cells were immediately processed for cell capture and library preparation using the Chromium Next GEM Single Cell 5′ Reagent Kits v2 (Dual Index) with Feature Barcode technology for Cell Surface Protein & Immune Receptor Mapping (CG000330 Rev D) according to the manufacture's instruction. Paired-end sequencing was performed with the NovaSeq 6000 (Illumina) using an S1 Reagent Kit version 1 (100 cycles) at the Genomics Facility Basel (28 nucleotides for the cell barcode and unique molecular identifier, 8 for the sample index, and 91 for the transcript read).

The mRNA data was mapped to the mm10 genome using the STARsolo framework (STAR version 2.7.10a).[105] Gene quantification made use of a custom GFT file based on the ensembl gene annotation (v.102). The cell barcode whitelist used for STAR is the file 737K-august-2016.txt provided by 10× Genomics. Other non-standard command line options for STAR included "–outFilterType BySJout –outFilterMultimapNmax 10 –outSAMmultNmax 1 –outSAMtype BAMSortedByCoordinate –outSAMunmapped Within –soloType CB_UMI_Simple –soloStrand Reverse –outFilterScoreMin 30 –soloCBmatchWLtype 1MM_multi_Nbase_pseudocounts –soloUMIlen 10 –soloUMIfiltering MultiGeneUMI_CR –soloUMIdedup 1MM_CR –soloCellFilter None –soloMultiMappers EM –soloBarcodeReadLength 0 –soloCBstart 1 –soloCBlen 16 –soloUMIstart 17 –soloFeatures Gene –outSAMattributes NH HI AS nM CR CY UR UY GX GN CB UB." Empty droplets were filtered with DropletUtils: emptyDrops (v.1.18.1)[106] with lower threshold set to 100.

The cDNA fastq files for cell surface hashtag data (ADT+HTO) were first trimmed with the tool fastp (v.0.20.1)[107] and command line options "-b 15 -f 10 -t 0 -A -Q -G –L." After cDNA trimming, cell surface hastag data were mapped and quantified with STARsolo, by using the same cell barcode whitelist as for GEX data. The genome index was built from the hashtag sequences as well as the GTF file used for quantification. Additional command line options included "–outSAMtype BAM SortedByCoordinate –outSAMunmapped Within –soloType CB_UMI_Simple –soloStrand Forward –soloCBmatchWLtype 1MM_multi_Nbase_pseudocounts –soloUMIlen 10 –soloUMIfiltering MultiGeneUMI_CR –soloUMIdedup 1MM_CR –soloCellFilter None –soloBarcodeReadLength 0 –soloCBstart 1 –soloCBlen 16 –soloUMIstart 17 –soloFeatures Gene." VDJ data were processed for T cell receptors with 10× Genomics Cell Ranger vdj (v.7.0.0). TCR sequencing and gene expression data were intersected, and cells were additionally filtered for high-confidence TRA and TRB chains. For the 12 samples, non-expressed genes were removed and scaling normalization was performed using batchelor:multiBatchNorm (v.1.18.1)[108] with mulitplexed sample name as batch factor. The cell cycle phase was assigned with tricycle (v.1.10.0).[109] The top 2,000 highly variable genes (HVGs) were selected with scran:modelGeneVar (v.1.30.2)[110] using the multiplexed sample name as blocking variable

and not considering B and T cell receptor variable genes. Batch correction on multiplexed sample names was performed with batchelor:-fastMNN on 15 neighbors, 50 dimensions, and the selected HVGs. For visualization, tSNE was performed with scater:runTSNE (v.1.30.1)[111] on the batch-corrected coordinates.

The cells were clustered on the batch-corrected coordinates with scran:buildSNNGraph with 15 neighbors, type "rank" weighting scheme, and igraph:cluster_louvain (v.2.1.1)[112] with resolution 0.6. Top markers per cluster were retrieved as high median AUC values obtained from scran:scoreMarkers with blocking factor the multiplexed sample names.

Cluster consisting of mostly cycling cells was removed, and normalization, dimensionality reduction, batch correction, and clustering were repeated as described above. Differential expression analysis was performed per cluster on pseudobulk samples with edgeR (v.4.0.16).[107]

Groups with less than 20 cells were removed, genes were filtered by expression with edgeR:filterByExpr, and scaling factors were computed with edgeR:calcNormFactors. The model was fitted with edgeR:glmQLFit, and contrasts were tested with edgeR:glmQLFTest. p values were adjusted for multiple testing with Benjamini-Hochberg (BH) procedure with edgeR:topTags. GSEA was performed on hallmark collection MH from MSigDb (v2023.2) with limma:camera (3.58.1)[113] using inter-gene correlation set to 0.01.

In vivo IFNAR blockade and NK cell depletion

For IFNAR blockade, mice were given 1 mg of anti-IFNAR monoclonal antibody (MAR-1-5A3, BioXcell) i.v. together with viral vectors in the same syringe. Control groups were administered 1 mg of isotype control antibody (MOPC-21, BioXcell) instead. For transfer experiments involving OT-1x*Ifnar*$^{-/-}$ cells, NK cells were depleted by administration of 300 μg of anti-NK.1.1 (PK136, BioXcell) monoclonal antibody to mice on day −1 and on day 1 of vector immunization, as described in Crouse et al.[75] and Xu et al.[76]

Adoptive T cell transfer

For OT-1 cell transfer, single-cell suspensions were prepared from spleens of naive OT-1 or OT-1x*Ifnar*$^{-/-}$ donor mice. Purification of CD8 T cells was performed with magnetic-activated cell sorting (naive CD8 T + T cell Isolation Kit, mouse, Miltenyi Biotec). The purity (>90%) was checked before transfer by FACS, and then cells were administered i.v. into the tail vein of syngeneic WT C57BL/6J recipients at a dose of 2,000 cells/mouse or 2E+06 cells/mouse for the experiments described in Figures 4 and 5, respectively. Transferred OT-1 populations expressed the congenic marker CD45.1 for differentiation from the recipient's endogenous cells.

Immunohistochemistry

For immunofluorescence analysis of spleen sections, OT-1 cells were magnetic cell separation (MACS)-purified (Miltenyi Biotec, naive CD8+ T Cell Isolation Kit, mouse) from the spleens of naive donor mice and 2E+06 cells were injected i.v. into the tail vein of wild-type recipient mice followed by vector immunization 2 days later. Animals were sacrificed at the indicated time points, and spleens were fixed in 1% PFA in PBS, infiltrated with 30% sucrose, and then embedded and frozen in OCT compound (Tissue-Tek, Sakura Finetek Europe). Tissue sections were stained for IFN-α, GFP, and CD45.1 as described previously.[114] In brief, cryostat sections were collected on Superfrost Plus Slides (Fisher Scientific), air dried, and preincubated with blocking solution (bovine serum albumin with mouse and chicken serum [Sigma] in 0.1% Triton/PBS). Then they were incubated overnight at 4°C with anti-IFN-α antibody (PBL, #32100-1) in 0.1% Triton/PBS. After washing with 0.1% Triton/PBS, anti-rabbit Alexa Fluor 647-conjugated (Life, #A31573) secondary antibody was added for 2 h at room temperature in 0.1% Triton/PBS. After an additional wash, tissue sections were incubated with Dako REAL peroxidase-blocking solution (Dako, K0672) to inactivate endogenous peroxidases and blocked to minimize unspecific binding (PBS supplemented with 2.5% goat serum). Sections were then incubated for 1 h with a chicken anti-GFP primary antibody (ICL, #CGFP-45ALY-Z). To visualize the specific signal, anti-chicken HRP-conjugated antibody (Jackson Immuno Research, #103-035-155) followed by amplification with TSA Vivid 520 (Tocris,7534) was used as secondary system. Finally, after a brief blocking step with mouse serum, sections were incubated with a PE-conjugated anti-CD45.1 antibody (BioLegend, #102707) and nuclei were stained with DAPI (Invitrogen, D1306). Slides were mounted in Fluoromount aqueous mounting medium (Sigma-Aldrich, F4680) for imaging. Stained sections were scanned at a resolution of 0.221 μm/pixel using the Panoramic 250 FLASH II Whole Slide Scanner (3DHISTECH). For samples stained for LCMV nucleoprotein, sections were incubated with anti-LCMV-NP antibody (clone VL-4, rat serum) in PBS containing 0.1% Triton X-100 together with the anti-IFN-α antibody.

Images were analyzed in Visiopharm (v.2025.2, Visiopharm, Denmark). For each marker, a U-Net based deep-learning classifier was trained and each cell labeled according to its marker expression profile. Total tissue area, cell counts, and per-cell marker positivity were quantified. For cell-cell contact analysis, CD45.1^{+} cell outlines were detected and expanded by 5 μm. U-Net-based classifiers were then applied to quantify the overlap of vector^{+} IFN-α^{+} and vector^{+} IFN-α^{-} structures with the extended CD45.1 outlines.

Statistical analysis

Statistical analyses were performed using GraphPad Prism v.9.0 (GraphPad Software). Pairwise comparisons were performed using two-tailed unpaired Student's t tests. For comparisons across multiple groups, one-way ANOVA followed by Tukey's post hoc test or two-way ANOVA with Bonferroni's post-test was applied as reported in the figure legend. Data are reported as mean ± standard error of the mean (SEM), unless otherwise specified. $p < 0.05$ (*) was considered statistically significant and $p < 0.01$ (**) as highly significant. Not statistically significant differences ($p > 0.05$) are not indicated in the figures unless specified.

DATA AND CODE AVAILABILITY

Raw data of the experimental results reported in this study have been deposited with Zenodo and are publicly available as of the date of publication under the https://doi.org/10.5281/zenodo.17055489. scRNA-seq raw data are available in the NCBI GEO database under accession number GSE317032.

ACKNOWLEDGMENTS

We wish to thank Karsten Stauffer for outstanding animal handling and care, Min Lu and Karen Cornille for excellent technical support, Christian Beisel and Mirjam Feldkamp from the Genomics Facility of the University of Basel and D-BSSE of ETH Zurich for single-cell RNA-seq library preparation and next-generation RNA sequencing, Cynthia Saadi for assistance with immunohistochemistry, Morgane Hilpert with the entire DBM flow cytometry core facility for expert cell sorting, sciCORE (http://scicore.unibas.ch/) scientific computing center at University of Basel, and the entire Experimental Virology group for helpful discussions. This project has received funding from the European Union's Horizon 2020 research and innovation programme under the Marie Skłodowska-Curie grant agreement no. 812915 and from the Swiss National Science Foundation (no. 310030_185318/1 to D.D.P.).

AUTHOR CONTRIBUTIONS

M.C., R.A., A.-F.M., T.A.-M, D.F., I.W., I.V., D.M., G.Z., and D.D.P. designed the experiments. M.C., R.A, A.-F.M., T.A.-M, D.F., J.F., F.G., D.B., I.W., I.V., and M.K. conducted the experiments and acquired and analyzed the data. M.C., G.Z., and D.D.P. wrote the manuscript.

DECLARATION OF INTERESTS

D.D.P. is a founder, consultant, and shareholder of Hookipa Pharma Inc. commercializing arenavirus-based vector technology, and he as well as D.M. are listed as inventors on corresponding patents.

SUPPLEMENTAL INFORMATION

Supplemental information can be found online at https://doi.org/10.1016/j.omtn.2026.102852.

REFERENCES

1. Appay, V., Douek, D.C., and Price, D.A. (2008). CD8+ T cell efficacy in vaccination and disease. Nat. Med. *14*, 623–628. https://doi.org/10.1038/nm.f.1774.
2. Bassi, M.R., Kongsgaard, M., Steffensen, M.A., Fenger, C., Rasmussen, M., Skjødt, K., Finsen, B., Stryhn, A., Buus, S., Christensen, J.P., and Thomsen, A.R. (2015). CD8+ T cells complement antibodies in protecting against yellow fever virus. J. Immunol. *194*, 1141–1153. https://doi.org/10.4049/jimmunol.1402605.
3. Chatterjee, B., Deng, Y., Holler, A., Nunez, N., Azzi, T., Vanoaica, L.D., Müller, A., Zdimerova, H., Antsiferova, O., Zbinden, A., et al. (2019). CD8+ T cells retain protective functions despite sustained inhibitory receptor expression during Epstein-Barr virus infection in vivo. PLoS Pathog. *15*, e1007748. https://doi.org/10.1371/journal.ppat.1007748.
4. Sullivan, N.J., Hensley, L., Asiedu, C., Geisbert, T.W., Stanley, D., Johnson, J., Honko, A., Olinger, G., Bailey, M., Geisbert, J.B., et al. (2011). CD8+ cellular immunity mediates rAd5 vaccine protection against Ebola virus infection of nonhuman primates. Nat. Med. *17*, 1128–1131. https://doi.org/10.1038/nm.2447.
5. Mateo, M., Reynard, S., Pietrosemoli, N., Perthame, E., Journeaux, A., Noy, K., Germain, C., Carnec, X., Picard, C., Borges-Cardoso, V., et al. (2023). Rapid protection induced by a single-shot Lassa vaccine in male cynomolgus monkeys. Nat. Commun. *14*, 1352. https://doi.org/10.1038/s41467-023-37050-6.
6. Carrington, M., Nelson, G.W., Martin, M.P., Kissner, T., Vlahov, D., Goedert, J.J., Kaslow, R., Buchbinder, S., Hoots, K., and O'Brien, S.J. (1999). HLA and HIV-1: heterozygote advantage and B*35-Cw*04 disadvantage. Science *283*, 1748–1752. https://doi.org/10.1126/science.283.5408.1748.
7. Goulder, P.J., Phillips, R.E., Colbert, R.A., McAdam, S., Ogg, G., Nowak, M.A., Giangrande, P., Luzzi, G., Morgan, B., Edwards, A., et al. (1997). Late escape from an immunodominant cytotoxic T-lymphocyte response associated with progression to AIDS. Nat. Med. *3*, 212–217. https://doi.org/10.1038/nm0297-212.
8. McMahan, K., Yu, J., Mercado, N.B., Loos, C., Tostanoski, L.H., Chandrashekar, A., Liu, J., Peter, L., Atyeo, C., Zhu, A., et al. (2021). Correlates of protection against SARS-CoV-2 in rhesus macaques. Nature *590*, 630–634. https://doi.org/10.1038/s41586-020-03041-6.
9. Chandrashekar, A., Yu, J., McMahan, K., Jacob-Dolan, C., Liu, J., He, X., Hope, D., Anioke, T., Barrett, J., Chung, B., et al. (2022). Vaccine protection against the SARS-CoV-2 Omicron variant in macaques. Cell *185*, 1549–1555.e11. https://doi.org/10.1016/j.cell.2022.03.024.
10. Collier, A.R.Y., Yu, J., McMahan, K., Liu, J., Chandrashekar, A., Maron, J.S., Atyeo, C., Martinez, D.R., Ansel, J.L., Aguayo, R., et al. (2021). Differential Kinetics of Immune Responses Elicited by Covid-19 Vaccines. N. Engl. J. Med. *385*, 2010–2012. https://doi.org/10.1056/NEJMc2115596.
11. Pardieck, I.N., van der Sluis, T.C., van der Gracht, E.T.I., Veerkamp, D.M.B., Behr, F.M., van Duikeren, S., Beyrend, G., Rip, J., Nadafi, R., Beyranvand Nejad, E., et al. (2022). A third vaccination with a single T cell epitope confers protection in a murine model of SARS-CoV-2 infection. Nat. Commun. *13*, 3966. https://doi.org/10.1038/s41467-022-31721-6.
12. Ishii, H., Nomura, T., Yamamoto, H., Nishizawa, M., Thu Hau, T.T., Harada, S., Seki, S., Nakamura-Hoshi, M., Okazaki, M., Daigen, S., et al. (2022). Neutralizing-antibody-independent SARS-CoV-2 control correlated with intranasal-vaccine-induced CD8(+) T cell responses. Cell Rep. Med. *3*, 100520. https://doi.org/10.1016/j.xcrm.2022.100520.
13. Fumagalli, V., Rava, M., Marotta, D., Di Lucia, P., Bono, E.B., Giustini, L., De Leo, F., Casalgrandi, M., Monteleone, E., Mouro, V., et al. (2024). Antibody-independent protection against heterologous SARS-CoV-2 challenge conferred by prior infection or vaccination. Nat. Immunol. *25*, 633–643. https://doi.org/10.1038/s41590-024-01787-z.
14. Liu, L., Iketani, S., Guo, Y., Chan, J.F.W., Wang, M., Liu, L., Luo, Y., Chu, H., Huang, Y., Nair, M.S., et al. (2022). Striking antibody evasion manifested by the Omicron variant of SARS-CoV-2. Nature *602*, 676–681. https://doi.org/10.1038/s41586-021-04388-0.
15. Alter, G., Yu, J., Liu, J., Chandrashekar, A., Borducchi, E.N., Tostanoski, L.H., McMahan, K., Jacob-Dolan, C., Martinez, D.R., Chang, A., et al. (2021). Immunogenicity of Ad26.COV2.S vaccine against SARS-CoV-2 variants in humans. Nature *596*, 268–272. https://doi.org/10.1038/s41586-021-03681-2.
16. Liu, J., Chandrashekar, A., Sellers, D., Barrett, J., Jacob-Dolan, C., Lifton, M., McMahan, K., Sciacca, M., VanWyk, H., Wu, C., et al. (2022). Vaccines elicit highly conserved cellular immunity to SARS-CoV-2 Omicron. Nature *603*, 493–496. https://doi.org/10.1038/s41586-022-04465-y.
17. Uddback, I., Michalets, S.E., Saha, A., Mattingly, C., Kost, K.N., Williams, M.E., Lawrence, L.A., Hicks, S.L., Lowen, A.C., Ahmed, H., et al. (2024). Prevention of respiratory virus transmission by resident memory CD8(+) T cells. Nature *626*, 392–400. https://doi.org/10.1038/s41586-023-06937-1.
18. Bolinger, B., Sims, S., Swadling, L., O'Hara, G., de Lara, C., Baban, D., Saghal, N., Lee, L.N., Marchi, E., Davis, M., et al. (2015). Adenoviral Vector Vaccination Induces a Conserved Program of CD8(+) T Cell Memory Differentiation in Mouse and Man. Cell Rep. *13*, 1578–1588. https://doi.org/10.1016/j.celrep.2015.10.034.
19. Flatz, L., Hegazy, A.N., Bergthaler, A., Verschoor, A., Claus, C., Fernandez, M., Gattinoni, L., Johnson, S., Kreppel, F., Kochanek, S., et al. (2010). Development of replication-defective lymphocytic choriomeningitis virus vectors for the induction of potent CD8+ T cell immunity. Nat. Med. *16*, 339–345. https://doi.org/10.1038/nm.2104.
20. Wang, S., Liang, B., Wang, W., Li, L., Feng, N., Zhao, Y., Wang, T., Yan, F., Yang, S., and Xia, X. (2023). Viral vectored vaccines: design, development, preventive and therapeutic applications in human diseases. Signal Transduct. Target. Ther. *8*, 149. https://doi.org/10.1038/s41392-023-01408-5.
21. Johnson, J.E., Nasar, F., Coleman, J.W., Price, R.E., Javadian, A., Draper, K., Lee, M., Reilly, P.A., Clarke, D.K., Hendry, R.M., and Udem, S.A. (2007). Neurovirulence properties of recombinant vesicular stomatitis virus vectors in non-human primates. Virology *360*, 36–49. https://doi.org/10.1016/j.virol.2006.10.026.
22. Huttner, A., Dayer, J.A., Yerly, S., Combescure, C., Auderset, F., Desmeules, J., Eickmann, M., Finckh, A., Goncalves, A.R., Hooper, J.W., et al. (2015). The effect

of dose on the safety and immunogenicity of the VSV Ebola candidate vaccine: a randomised double-blind, placebo-controlled phase 1/2 trial. Lancet Infect. Dis. *15*, 1156–1166. https://doi.org/10.1016/S1473-3099(15)00154-1.

23. Publicover, J., Ramsburg, E., and Rose, J.K. (2005). A single-cycle vaccine vector based on vesicular stomatitis virus can induce immune responses comparable to those generated by a replication-competent vector. J. Virol. *79*, 13231–13238. https://doi.org/10.1128/JVI.79.21.13231-13238.2005.
24. Kapadia, S.U., Simon, I.D., and Rose, J.K. (2008). SARS vaccine based on a replication-defective recombinant vesicular stomatitis virus is more potent than one based on a replication-competent vector. Virology *376*, 165–172. https://doi.org/10.1016/j.virol.2008.03.002.
25. Majid, A.M., Ezelle, H., Shah, S., and Barber, G.N. (2006). Evaluating replication-defective vesicular stomatitis virus as a vaccine vehicle. J. Virol. *80*, 6993–7008. https://doi.org/10.1128/JVI.00365-06.
26. Quinn, K.M., Zak, D.E., Costa, A., Yamamoto, A., Kastenmuller, K., Hill, B.J., Lynn, G.M., Darrah, P.A., Lindsay, R.W.B., Wang, L., et al. (2015). Antigen expression determines adenoviral vaccine potency independent of IFN and STING signaling. J. Clin. Investig. *125*, 1129–1146. https://doi.org/10.1172/JCI78280.
27. Rhee, E.G., Blattman, J.N., Kasturi, S.P., Kelley, R.P., Kaufman, D.R., Lynch, D.M., La Porte, A., Simmons, N.L., Clark, S.L., Pulendran, B., et al. (2011). Multiple innate immune pathways contribute to the immunogenicity of recombinant adenovirus vaccine vectors. J. Virol. *85*, 315–323. https://doi.org/10.1128/JVI.01597-10.
28. Nielsen, K.N., Steffensen, M.A., Christensen, J.P., and Thomsen, A.R. (2014). Priming of CD8 T cells by adenoviral vectors is critically dependent on B7 and dendritic cells but only partially dependent on CD28 ligation on CD8 T cells. J. Immunol. *193*, 1223–1232. https://doi.org/10.4049/jimmunol.1400197.
29. Ku, M.W., Authié, P., Nevo, F., Souque, P., Bourgine, M., Romano, M., Charneau, P., and Majlessi, L. (2021). Lentiviral vector induces high-quality memory T cells via dendritic cells transduction. Commun. Biol. *4*, 713. https://doi.org/10.1038/s42003-021-02251-6.
30. Finn, J.D., Bassett, J., Millar, J.B., Grinshtein, N., Yang, T.C., Parsons, R., Evelegh, C., Wan, Y., Parks, R.J., and Bramson, J.L. (2009). Persistence of transgene expression influences CD8+ T-cell expansion and maintenance following immunization with recombinant adenovirus. J. Virol. *83*, 12027–12036. https://doi.org/10.1128/JVI.00593-09.
31. Wennier, S.T., Brinkmann, K., Steinhäußer, C., Mayländer, N., Mnich, C., Wielert, U., Dirmeier, U., Hausmann, J., Chaplin, P., and Steigerwald, R. (2013). A novel naturally occurring tandem promoter in modified vaccinia virus ankara drives very early gene expression and potent immune responses. PLoS One *8*, e73511. https://doi.org/10.1371/journal.pone.0073511.
32. Baur, K., Brinkmann, K., Schweneker, M., Pätzold, J., Meisinger-Henschel, C., Hermann, J., Steigerwald, R., Chaplin, P., Suter, M., and Hausmann, J. (2010). Immediate-early expression of a recombinant antigen by modified vaccinia virus ankara breaks the immunodominance of strong vector-specific B8R antigen in acute and memory CD8 T-cell responses. J. Virol. *84*, 8743–8752. https://doi.org/10.1128/JVI.00604-10.
33. Becker, P., Nörder, M., Weissmann, S., Ljapoci, R., Erfle, V., Drexler, I., and Guzmán, C. (2014). Gene Expression Driven by a Strong Viral Promoter in MVA Increases Vaccination Efficiency by Enhancing Antibody Responses and Unmasking CD8(+) T Cell Epitopes. Vaccines (Basel) *2*, 581–600. https://doi.org/10.3390/vaccines2030581.
34. Palacio, N., Dangi, T., Chung, Y.R., Wang, Y., Loredo-Varela, J.L., Zhang, Z., and Penaloza-MacMaster, P. (2020). Early type I IFN blockade improves the efficacy of viral vaccines. J. Exp. Med. *217*, e20191220. https://doi.org/10.1084/jem.20191220.
35. Frenz, T., Waibler, Z., Hofmann, J., Hamdorf, M., Lantermann, M., Reizis, B., Tovey, M.G., Aichele, P., Sutter, G., and Kalinke, U. (2010). Concomitant type I IFN receptor-triggering of T cells and of DC is required to promote maximal modified vaccinia virus Ankara-induced T-cell expansion. Eur. J. Immunol. *40*, 2769–2777. https://doi.org/10.1002/eji.201040453.
36. Zhong, C., Liu, F., Hajnik, R.J., Yao, L., Chen, K., Wang, M., Liang, Y., Sun, J., Soong, L., Hou, W., and Hu, H. (2021). Type I Interferon Promotes Humoral Immunity in Viral Vector Vaccination. J. Virol. *95*, e0092521. https://doi.org/10.1128/JVI.00925-21.
37. Waibler, Z., Anzaghe, M., Frenz, T., Schwantes, A., Pöhlmann, C., Ludwig, H., Palomo-Otero, M., Alcamí, A., Sutter, G., and Kalinke, U. (2009). Vaccinia virus-mediated inhibition of type I interferon responses is a multifactorial process involving the soluble type I interferon receptor B18 and intracellular components. J. Virol. *83*, 1563–1571. https://doi.org/10.1128/JVI.01617-08.
38. Nidetz, N.F., Gallagher, T.M., and Wiethoff, C.M. (2018). Inhibition of type I interferon responses by adenovirus serotype-dependent Gas6 binding. Virology *515*, 150–157. https://doi.org/10.1016/j.virol.2017.12.016.
39. Dai, P., Wang, W., Cao, H., Avogadri, F., Dai, L., Drexler, I., Joyce, J.A., Li, X.D., Chen, Z., Merghoub, T., et al. (2014). Modified vaccinia virus Ankara triggers type I IFN production in murine conventional dendritic cells via a cGAS/STING-mediated cytosolic DNA-sensing pathway. PLoS Pathog. *10*, e1003989. https://doi.org/10.1371/journal.ppat.1003989.
40. Choi, H.J., Park, A., Kang, S., Lee, E., Lee, T.A., Ra, E.A., Lee, J., Lee, S., and Park, B. (2018). Human cytomegalovirus-encoded US9 targets MAVS and STING signaling to evade type I interferon immune responses. Nat. Commun. *9*, 125. https://doi.org/10.1038/s41467-017-02624-8.
41. Plesa, G., McKenna, P.M., Schnell, M.J., and Eisenlohr, L.C. (2006). Immunogenicity of cytopathic and noncytopathic viral vectors. J. Virol. *80*, 6259–6266. https://doi.org/10.1128/JVI.00084-06.
42. Hangartner, L., Zinkernagel, R.M., and Hengartner, H. (2006). Antiviral antibody responses: the two extremes of a wide spectrum. Nat. Rev. Immunol. *6*, 231–243. https://doi.org/10.1038/nri1783.
43. Lefrancois, L., and Lyles, D.S. (1982). The interaction of antibody with the major surface glycoprotein of vesicular stomatitis virus. II. Monoclonal antibodies of nonneutralizing and cross-reactive epitopes of Indiana and New Jersey serotypes. Virology *121*, 168–174. https://doi.org/10.1016/0042-6822(82)90126-x.
44. Gobet, R., Cerny, A., Rüedi, E., Hengartner, H., and Zinkernagel, R.M. (1988). The role of antibodies in natural and acquired resistance of mice to vesicular stomatitis virus. Exp. Cell Biol. *56*, 175–180. https://doi.org/10.1159/000163477.
45. Zinkernagel, R.M., Cooper, S., Chambers, J., Lazzarini, R.A., Hengartner, H., and Arnheiter, H. (1990). Virus-induced autoantibody response to a transgenic viral antigen. Nature *345*, 68–71. https://doi.org/10.1038/345068a0.
46. Pinschewer, D.D., Perez, M., Jeetendra, E., Bächi, T., Horvath, E., Hengartner, H., Whitt, M.A., de la Torre, J.C., and Zinkernagel, R.M. (2004). Kinetics of protective antibodies are determined by the viral surface antigen. J. Clin. Investig. *114*, 988–993. https://doi.org/10.1172/JCI22374.
47. Henao-Restrepo, A.M., Longini, I.M., Egger, M., Dean, N.E., Edmunds, W.J., Camacho, A., Carroll, M.W., Doumbia, M., Draguez, B., Duraffour, S., et al. (2015). Efficacy and effectiveness of an rVSV-vectored vaccine expressing Ebola surface glycoprotein: interim results from the Guinea ring vaccination cluster-randomised trial. Lancet *386*, 857–866. https://doi.org/10.1016/S0140-6736(15)61117-5.
48. Cross, R.W., Xu, R., Matassov, D., Hamm, S., Latham, T.E., Gerardi, C.S., Nowak, R.M., Geisbert, J.B., Ota-Setlik, A., Agans, K.N., et al. (2020). Quadrivalent VesiculoVax vaccine protects nonhuman primates from viral-induced hemorrhagic fever and death. J. Clin. Investig. *130*, 539–551. https://doi.org/10.1172/JCI131958.
49. Sulis, G., Peebles, A., and Basta, N.E. (2023). Lassa fever vaccine candidates: A scoping review of vaccine clinical trials. Trop. Med. Int. Health *28*, 420–431. https://doi.org/10.1111/tmi.13876.
50. Geisbert, T.W., Jones, S., Fritz, E.A., Shurtleff, A.C., Geisbert, J.B., Liebscher, R., Grolla, A., Ströher, U., Fernando, L., Daddario, K.M., et al. (2005). Development of a new vaccine for the prevention of Lassa fever. PLoS Med. *2*, e183. https://doi.org/10.1371/journal.pmed.0020183.
51. Marzi, A., Engelmann, F., Feldmann, F., Haberthur, K., Shupert, W.L., Brining, D., Scott, D.P., Geisbert, T.W., Kawaoka, Y., Katze, M.G., et al. (2013). Antibodies are necessary for rVSV/ZEBOV-GP-mediated protection against lethal Ebola virus challenge in nonhuman primates. Proc. Natl. Acad. Sci. USA *110*, 1893–1898. https://doi.org/10.1073/pnas.1209591110.
52. Her, L.S., Lund, E., and Dahlberg, J.E. (1997). Inhibition of Ran guanosine triphosphatase-dependent nuclear transport by the matrix protein of vesicular stomatitis virus. Science *276*, 1845–1848. https://doi.org/10.1126/science.276.5320.1845.

53. von Kobbe, C., van Deursen, J.M., Rodrigues, J.P., Sitterlin, D., Bachi, A., Wu, X., Wilm, M., Carmo-Fonseca, M., and Izaurralde, E. (2000). Vesicular stomatitis virus matrix protein inhibits host cell gene expression by targeting the nucleoporin Nup98. Mol. Cell *6*, 1243–1252. https://doi.org/10.1016/s1097-2765(00)00120-9.

54. Faria, P.A., Chakraborty, P., Levay, A., Barber, G.N., Ezelle, H.J., Enninga, J., Arana, C., van Deursen, J., and Fontoura, B.M.A. (2005). VSV disrupts the Rae1/mrnp41 mRNA nuclear export pathway. Mol. Cell *17*, 93–102. https://doi.org/10.1016/j.molcel.2004.11.023.

55. Kopecky, S.A., and Lyles, D.S. (2003). The cell-rounding activity of the vesicular stomatitis virus matrix protein is due to the induction of cell death. J. Virol. *77*, 5524–5528. https://doi.org/10.1128/jvi.77.9.5524-5528.2003.

56. Gaddy, D.F., and Lyles, D.S. (2005). Vesicular stomatitis viruses expressing wild-type or mutant M proteins activate apoptosis through distinct pathways. J. Virol. *79*, 4170–4179. https://doi.org/10.1128/JVI.79.7.4170-4179.2005.

57. Schreiber, L.M., Urbiola, C., Das, K., Spiesschaert, B., Kimpel, J., Heinemann, F., Stierstorfer, B., Müller, P., Petersson, M., Erlmann, P., et al. (2019). The lytic activity of VSV-GP treatment dominates the therapeutic effects in a syngeneic model of lung cancer. Br. J. Cancer *121*, 647–658. https://doi.org/10.1038/s41416-019-0574-7.

58. Pol, J.G., Zhang, L., Bridle, B.W., Stephenson, K.B., Rességuier, J., Hanson, S., Chen, L., Kazdhan, N., Bramson, J.L., Stojdl, D.F., et al. (2014). Maraba virus as a potent oncolytic vaccine vector. Mol. Ther. *22*, 420–429. https://doi.org/10.1038/mt.2013.249.

59. Ahmed, M., McKenzie, M.O., Puckett, S., Hojnacki, M., Poliquin, L., and Lyles, D.S. (2003). Ability of the matrix protein of vesicular stomatitis virus to suppress beta interferon gene expression is genetically correlated with the inhibition of host RNA and protein synthesis. J. Virol. *77*, 4646–4657. https://doi.org/10.1128/jvi.77.8.4646-4657.2003.

60. Hanika, A., Larisch, B., Steinmann, E., Schwegmann-Weßels, C., Herrler, G., and Zimmer, G. (2005). Use of influenza C virus glycoprotein HEF for generation of vesicular stomatitis virus pseudotypes. J. Gen. Virol. *86*, 1455–1465. https://doi.org/10.1099/vir.0.80788-0.

61. Sanchez, S., Palacio, N., Dangi, T., Ciucci, T., and Penaloza-MacMaster, P. (2021). Fractionating a COVID-19 Ad5-vectored vaccine improves virus-specific immunity. Sci. Immunol. *6*, eabi8635. https://doi.org/10.1126/sciimmunol.abi8635.

62. Zinkernagel, R.M. (2000). Localization dose and time of antigens determine immune reactivity. Semin. Immunol. *12*, 163–344. https://doi.org/10.1006/smim.2000.0253.

63. Hoffmann, M., Wu, Y.J., Gerber, M., Berger-Rentsch, M., Heimrich, B., Schwemmle, M., and Zimmer, G. (2010). Fusion-active glycoprotein G mediates the cytotoxicity of vesicular stomatitis virus M mutants lacking host shut-off activity. J. Gen. Virol. *91*, 2782–2793. https://doi.org/10.1099/vir.0.023978-0.

64. Forbes, E.K., Biswas, S., Collins, K.A., Gilbert, S.C., Hill, A.V.S., and Draper, S.J. (2011). Combining liver- and blood-stage malaria viral-vectored vaccines: investigating mechanisms of CD8+ T cell interference. J. Immunol. *187*, 3738–3750. https://doi.org/10.4049/jimmunol.1003783.

65. Condotta, S.A., Richer, M.J., Badovinac, V.P., and Harty, J.T. (2012). Probing CD8 T cell responses with Listeria monocytogenes infection. Adv. Immunol. *113*, 51–80. https://doi.org/10.1016/B978-0-12-394590-7.00005-1.

66. Olson, J.A., McDonald-Hyman, C., Jameson, S.C., and Hamilton, S.E. (2013). Effector-like CD8(+) T cells in the memory population mediate potent protective immunity. Immunity *38*, 1250–1260. https://doi.org/10.1016/j.immuni.2013.05.009.

67. Evrard, M., Wynne-Jones, E., Peng, C., Kato, Y., Christo, S.N., Fonseca, R., Park, S.L., Burn, T.N., Osman, M., Devi, S., et al. (2022). Sphingosine 1-phosphate receptor 5 (S1PR5) regulates the peripheral retention of tissue-resident lymphocytes. J. Exp. Med. *219*, e20210116. https://doi.org/10.1084/jem.20210116.

68. Omilusik, K.D., Best, J.A., Yu, B., Goossens, S., Weidemann, A., Nguyen, J.V., Seuntjens, E., Stryjewska, A., Zweier, C., Roychoudhuri, R., et al. (2015). Transcriptional repressor ZEB2 promotes terminal differentiation of CD8+ effector and memory T cell populations during infection. J. Exp. Med. *212*, 2027–2039. https://doi.org/10.1084/jem.20150194.

69. Kallert, S.M., Darbre, S., Bonilla, W.V., Kreutzfeldt, M., Page, N., Müller, P., Kreuzaler, M., Lu, M., Favre, S., Kreppel, F., et al. (2017). Replicating viral vector platform exploits alarmin signals for potent CD8(+) T cell-mediated tumour immunotherapy. Nat. Commun. *8*, 15327. https://doi.org/10.1038/ncomms15327.

70. Marquis, K.A., Becker, R.L., Weiss, A.N., Morris, M.C., and Ferran, M.C. (2020). The VSV matrix protein inhibits NF-kappaB and the interferon response independently in mouse L929 cells. Virology *548*, 117–123. https://doi.org/10.1016/j.virol.2020.06.013.

71. Rieder, M., and Conzelmann, K.K. (2009). Rhabdovirus evasion of the interferon system. J. Interferon Cytokine Res. *29*, 499–509. https://doi.org/10.1089/jir.2009.0068.

72. Rodrigo, W.W.S.I., Ortiz-Riaño, E., Pythoud, C., Kunz, S., de la Torre, J.C., and Martínez-Sobrido, L. (2012). Arenavirus nucleoproteins prevent activation of nuclear factor kappa B. J. Virol. *86*, 8185–8197. https://doi.org/10.1128/JVI.07240-11.

73. Pythoud, C., Rodrigo, W.W.S.I., Pasqual, G., Rothenberger, S., Martínez-Sobrido, L., de la Torre, J.C., and Kunz, S. (2012). Arenavirus nucleoprotein targets interferon regulatory factor-activating kinase IKKepsilon. J. Virol. *86*, 7728–7738. https://doi.org/10.1128/JVI.00187-12.

74. Keppler, S.J., Theil, K., Vucikuja, S., and Aichele, P. (2009). Effector T-cell differentiation during viral and bacterial infections: Role of direct IL-12 signals for cell fate decision of CD8(+) T cells. Eur. J. Immunol. *39*, 1774–1783. https://doi.org/10.1002/eji.200839093.

75. Crouse, J., Bedenikovic, G., Wiesel, M., Ibberson, M., Xenarios, I., Von Laer, D., Kalinke, U., Vivier, E., Jonjic, S., and Oxenius, A. (2014). Type I interferons protect T cells against NK cell attack mediated by the activating receptor NCR1. Immunity *40*, 961–973. https://doi.org/10.1016/j.immuni.2014.05.003.

76. Xu, H.C., Grusdat, M., Pandyra, A.A., Polz, R., Huang, J., Sharma, P., Deenen, R., Köhrer, K., Rahbar, R., Diefenbach, A., et al. (2014). Type I interferon protects antiviral CD8+ T cells from NK cell cytotoxicity. Immunity *40*, 949–960. https://doi.org/10.1016/j.immuni.2014.05.004.

77. van Boxel-Dezaire, A.H.H., Rani, M.R.S., and Stark, G.R. (2006). Complex modulation of cell type-specific signaling in response to type I interferons. Immunity *25*, 361–372. https://doi.org/10.1016/j.immuni.2006.08.014.

78. Broomfield, B.J., Tan, C.W., Qin, R.Z., Abberger, H., Duckworth, B.C., Alvarado, C., Dalit, L., Lee, C.L., Shandre Mugan, R., Mazrad, Z.A.I., et al. (2025). Transient inhibition of type I interferon enhances CD8+ T cell stemness and vaccine protection. J. Exp. Med. *222*, e20241148. https://doi.org/10.1084/jem.20241148.

79. Kolumam, G.A., Thomas, S., Thompson, L.J., Sprent, J., and Murali-Krishna, K. (2005). Type I interferons act directly on CD8 T cells to allow clonal expansion and memory formation in response to viral infection. J. Exp. Med. *202*, 637–650. https://doi.org/10.1084/jem.20050821.

80. Starbeck-Miller, G.R., Xue, H.H., and Harty, J.T. (2014). IL-12 and type I interferon prolong the division of activated CD8 T cells by maintaining high-affinity IL-2 signaling in vivo. J. Exp. Med. *211*, 105–120. https://doi.org/10.1084/jem.20130901.

81. Wiesel, M., Crouse, J., Bedenikovic, G., Sutherland, A., Joller, N., and Oxenius, A. (2012). Type-I IFN drives the differentiation of short-lived effector CD8+ T cells in vivo. Eur. J. Immunol. *42*, 320–329. https://doi.org/10.1002/eji.201142091.

82. Thompson, L.J., Kolumam, G.A., Thomas, S., and Murali-Krishna, K. (2006). Innate inflammatory signals induced by various pathogens differentially dictate the IFN-I dependence of CD8 T cells for clonal expansion and memory formation. J. Immunol. *177*, 1746–1754. https://doi.org/10.4049/jimmunol.177.3.1746.

83. Keppler, S.J., and Aichele, P. (2011). Signal 3 requirement for memory CD8+ T-cell activation is determined by the infectious pathogen. Eur. J. Immunol. *41*, 3176–3186. https://doi.org/10.1002/eji.201141537.

84. Pichlmair, A., Schulz, O., Tan, C.P., Näslund, T.I., Liljeström, P., Weber, F., and Reis e Sousa, C. (2006). RIG-I-mediated antiviral responses to single-stranded RNA bearing 5'-phosphates. Science *314*, 997–1001. https://doi.org/10.1126/science.1132998.

85. Pipperger, L., Riepler, L., Kimpel, J., Siller, A., Stoitzner, P., Bánki, Z., and von Laer, D. (2021). Differential infection of murine and human dendritic cell subsets by oncolytic vesicular stomatitis virus variants. OncoImmunology *10*, 1959140. https://doi.org/10.1080/2162402X.2021.1959140.

86. Redondo, N., Madan, V., Alvarez, E., and Carrasco, L. (2015). Impact of Vesicular Stomatitis Virus M Proteins on Different Cellular Functions. PLoS One *10*, e0131137. https://doi.org/10.1371/journal.pone.0131137.

87. Stojdl, D.F., Lichty, B.D., tenOever, B.R., Paterson, J.M., Power, A.T., Knowles, S., Marius, R., Reynard, J., Poliquin, L., Atkins, H., et al. (2003). VSV strains with defects in their ability to shutdown innate immunity are potent systemic anti-cancer agents. Cancer Cell *4*, 263–275. https://doi.org/10.1016/s1535-6108(03)00241-1.

88. Fallet, B., Narr, K., Ertuna, Y.I., Remy, M., Sommerstein, R., Cornille, K., Kreutzfeldt, M., Page, N., Zimmer, G., Geier, F., et al. (2016). Interferon-driven deletion of antiviral B cells at the onset of chronic infection. Sci. Immunol. *1*, eaah6817. https://doi.org/10.1126/sciimmunol.aah6817.

89. Sammicheli, S., Kuka, M., Di Lucia, P., de Oya, N.J., De Giovanni, M., Fioravanti, J., Cristofani, C., Maganuco, C.G., Fallet, B., Ganzer, L., et al. (2016). Inflammatory monocytes hinder antiviral B cell responses. Sci. Immunol. *1*, eaah6789. https://doi.org/10.1126/sciimmunol.aah6789.

90. Moseman, E.A., Wu, T., de la Torre, J.C., Schwartzberg, P.L., and McGavern, D.B. (2016). Type I interferon suppresses virus-specific B cell responses by modulating CD8(+) T cell differentiation. Sci. Immunol. *1*, eaah3565. https://doi.org/10.1126/sciimmunol.aah3565.

91. Coughlan, L. (2020). Factors Which Contribute to the Immunogenicity of Non-replicating Adenoviral Vectored Vaccines. Front. Immunol. *11*, 909. https://doi.org/10.3389/fimmu.2020.00909.

92. Cao, W., Henry, M.D., Borrow, P., Yamada, H., Elder, J.H., Ravkov, E.V., Nichol, S.T., Compans, R.W., Campbell, K.P., and Oldstone, M.B. (1998). Identification of alpha-dystroglycan as a receptor for lymphocytic choriomeningitis virus and Lassa fever virus. Science *282*, 2079–2081. https://doi.org/10.1126/science.282.5396.2079.

93. Finkelshtein, D., Werman, A., Novick, D., Barak, S., and Rubinstein, M. (2013). LDL receptor and its family members serve as the cellular receptors for vesicular stomatitis virus. Proc. Natl. Acad. Sci. USA *110*, 7306–7311. https://doi.org/10.1073/pnas.1214441110.

94. Tober, R., Banki, Z., Egerer, L., Muik, A., Behmüller, S., Kreppel, F., Greczmiel, U., Oxenius, A., von Laer, D., and Kimpel, J. (2014). VSV-GP: a potent viral vaccine vector that boosts the immune response upon repeated applications. J. Virol. *88*, 4897–4907. https://doi.org/10.1128/JVI.03276-13.

95. Schwendinger, M., Thiry, G., De Vos, B., Leroux-Roels, G., Bruhwyler, J., Huygens, A., Ganeff, C., Buchinger, H., Orlinger, K.K., Pinschewer, D.D., et al. (2022). A Randomized Dose-Escalating Phase I Trial of a Replication-Deficient Lymphocytic Choriomeningitis Virus Vector-Based Vaccine Against Human Cytomegalovirus. J. Infect. Dis. *225*, 1399–1410. https://doi.org/10.1093/infdis/jiaa121.

96. Oxenius, A., Karrer, U., Zinkernagel, R.M., and Hengartner, H. (1999). IL-12 is not required for induction of type 1 cytokine responses in viral infections. J. Immunol. *162*, 965–973.

97. Kalia, V., Sarkar, S., Subramaniam, S., Haining, W.N., Smith, K.A., and Ahmed, R. (2010). Prolonged interleukin-2Ralpha expression on virus-specific CD8+ T cells favors terminal-effector differentiation in vivo. Immunity *32*, 91–103. https://doi.org/10.1016/j.immuni.2009.11.010.

98. Grob, P., Schijns, V.E., van den Broek, M.F., Cox, S.P., Ackermann, M., and Suter, M. (1999). Role of the individual interferon systems and specific immunity in mice in controlling systemic dissemination of attenuated pseudorabies virus infection. J. Virol. *73*, 4748–4754. https://doi.org/10.1128/JVI.73.6.4748-4754.1999.

99. Muller, U., Steinhoff, U., Reis, L.F., Hemmi, S., Pavlovic, J., Zinkernagel, R.M., and Aguet, M. (1994). Functional role of type I and type II interferons in antiviral defense. Science *264*, 1918–1921. https://doi.org/10.1126/science.8009221.

100. Hogquist, K.A., Jameson, S.C., Heath, W.R., Howard, J.L., Bevan, M.J., and Carbone, F.R. (1994). T cell receptor antagonist peptides induce positive selection. Cell *76*, 17–27. https://doi.org/10.1016/0092-8674(94)90169-4.

101. Flatz, L., Bergthaler, A., de la Torre, J.C., and Pinschewer, D.D. (2006). Recovery of an arenavirus entirely from RNA polymerase I/II-driven cDNA. Proc. Natl. Acad. Sci. USA *103*, 4663–4668. https://doi.org/10.1073/pnas.0600652103.

102. Battegay, M., Cooper, S., Althage, A., Bänziger, J., Hengartner, H., and Zinkernagel, R.M. (1991). Quantification of lymphocytic choriomeningitis virus with an immunological focus assay in 24- or 96-well plates. J. Virol. Methods *33*, 191–198. https://doi.org/10.1016/0166-0934(91)90018-u.

103. Charan, S., and Zinkernagel, R.M. (1986). Antibody mediated suppression of secondary IgM response in nude mice against vesicular stomatitis virus. J. Immunol. *136*, 3057–3061.

104. Pope, C., Kim, S.K., Marzo, A., Masopust, D., Williams, K., Jiang, J., Shen, H., and Lefrançois, L. (2001). Organ-specific regulation of the CD8 T cell response to Listeria monocytogenes infection. J. Immunol. *166*, 3402–3409. https://doi.org/10.4049/jimmunol.166.5.3402.

105. Dobin, A., Davis, C.A., Schlesinger, F., Drenkow, J., Zaleski, C., Jha, S., Batut, P., Chaisson, M., and Gingeras, T.R. (2013). STAR: ultrafast universal RNA-seq aligner. Bioinformatics *29*, 15–21. https://doi.org/10.1093/bioinformatics/bts635.

106. Lun, A.T.L., Riesenfeld, S., Andrews, T., Dao, T.P., Gomes, T.; participants in the 1st Human Cell Atlas Jamboree, and Marioni, J.C. (2019). EmptyDrops: distinguishing cells from empty droplets in droplet-based single-cell RNA sequencing data. Genome Biol. *20*, 63. https://doi.org/10.1186/s13059-019-1662-y.

107. Chen, Y., Chen, L., Lun, A.T.L., Baldoni, P.L., and Smyth, G.K. (2025). edgeR v4: powerful differential analysis of sequencing data with expanded functionality and improved support for small counts and larger datasets. Nucleic Acids Res. *53*, gkaf018. https://doi.org/10.1093/nar/gkaf018.

108. Haghverdi, L., Lun, A.T.L., Morgan, M.D., and Marioni, J.C. (2018). Batch effects in single-cell RNA-sequencing data are corrected by matching mutual nearest neighbors. Nat. Biotechnol. *36*, 421–427. https://doi.org/10.1038/nbt.4091.

109. Zheng, S.C., Stein-O'Brien, G., Augustin, J.J., Slosberg, J., Carosso, G.A., Winer, B., Shin, G., Bjornsson, H.T., Goff, L.A., and Hansen, K.D. (2022). Universal prediction of cell-cycle position using transfer learning. Genome Biol. *23*, 41. https://doi.org/10.1186/s13059-021-02581-y.

110. Lun, A.T.L., McCarthy, D.J., and Marioni, J.C. (2016). A step-by-step workflow for low-level analysis of single-cell RNA-seq data with Bioconductor. F1000Res. *5*, 2122. https://doi.org/10.12688/f1000research.9501.2.

111. McCarthy, D.J., Campbell, K.R., Lun, A.T.L., and Wills, Q.F. (2017). Scater: pre-processing, quality control, normalization and visualization of single-cell RNA-seq data in R. Bioinformatics *33*, 1179–1186. https://doi.org/10.1093/bioinformatics/btw777.

112. Csárdi, G., Nepusz, T., Müller, K., Horvát, S., Traag, V., Zanini, F., and Noom, D. (2024). Igraph for R: R Interface of the Igraph Library for Graph Theory and Network Analysis (v2.1.1) (Zenodo). https://doi.org/10.5281/zenodo.13964143.

113. Ritchie, M.E., Phipson, B., Wu, D., Hu, Y., Law, C.W., Shi, W., and Smyth, G.K. (2015). limma powers differential expression analyses for RNA-sequencing and microarray studies. Nucleic Acids Res. *43*, e47. https://doi.org/10.1093/nar/gkv007.

114. Marx, A.F., Kallert, S.M., Brunner, T.M., Villegas, J.A., Geier, F., Fixemer, J., Abreu-Mota, T., Reuther, P., Bonilla, W.V., Fadejeva, J., et al. (2023). The alarmin interleukin-33 promotes the expansion and preserves the stemness of Tcf-1(+) CD8(+) T cells in chronic viral infection. Immunity *56*, 813–828.e10. https://doi.org/10.1016/j.immuni.2023.01.029.

OMTN, Volume *37*

Supplemental information

Non-cytolytic re-engineering of a viral vaccine vector enables durable effector-memory T cell immunity by reinforcing type I IFN induction

Matias Ciancaglini, Robin Avanthay, Anna-Friederike Marx, Tiago Abreu-Mota, Davide Finozzi, Jonas Fixemer, Florian Geier, Dominik Burri, Ingrid Wagner, Ilena Vincenti, Mario Kreuzfeldt, Doron Merkler, Gert Zimmer, and Daniel D. Pinschewer

Supplementary figures

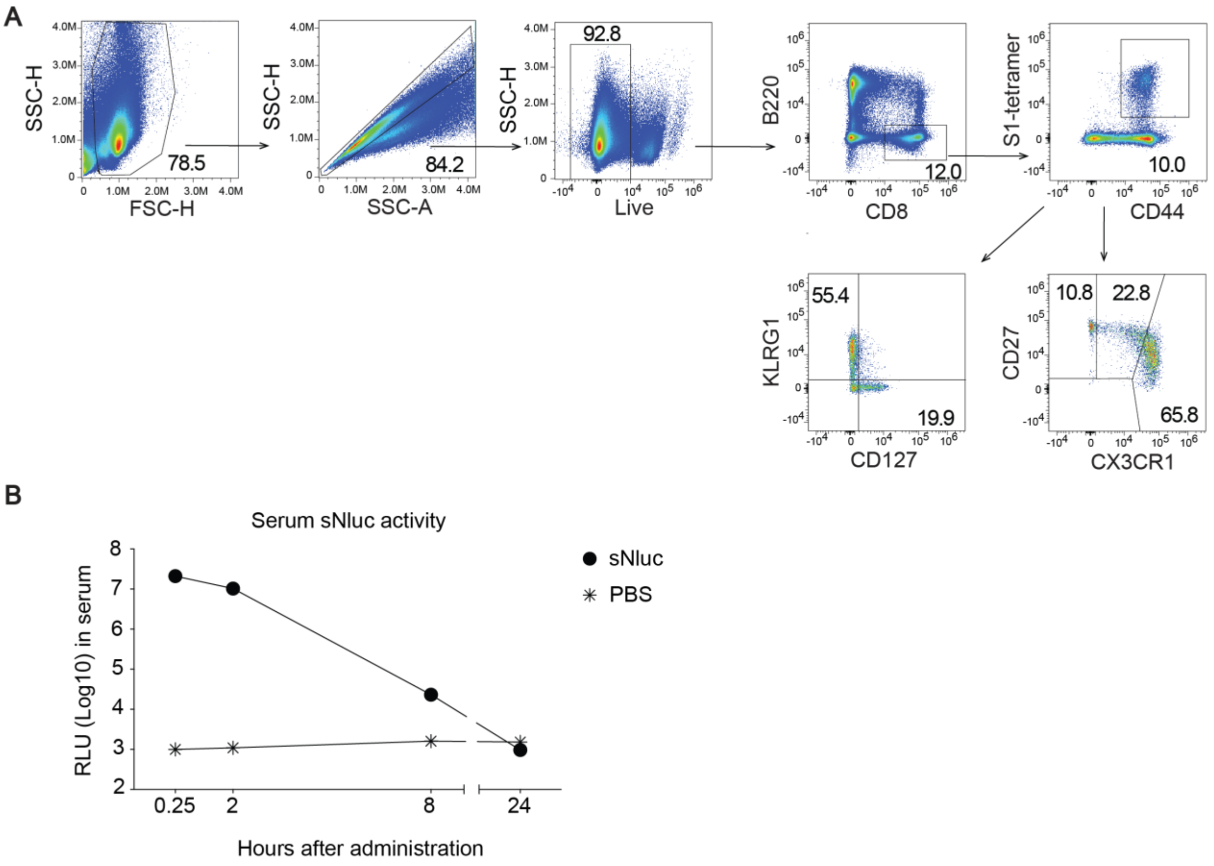

Figure S1. Gating strategy for epitope-specific CD8 T cells, and *in vivo* half-life of secreted nanoluciferase. (A) Gating strategy for the enumeration and characterization of tetramer-binding CD8$^+$ T cells. (B) Mice were administered cell culture supernatant containing 1E+08 RLU of sNluc or PBS control i.v. and blood was collected to determine luciferase activity in serum over time. Symbols represent the mean±SEM of 4 mice per group. Half-life of sNluc in serum was 0.7 hours, as determined by one-phase decay analysis with non-linear regression using GraphPad Prism.

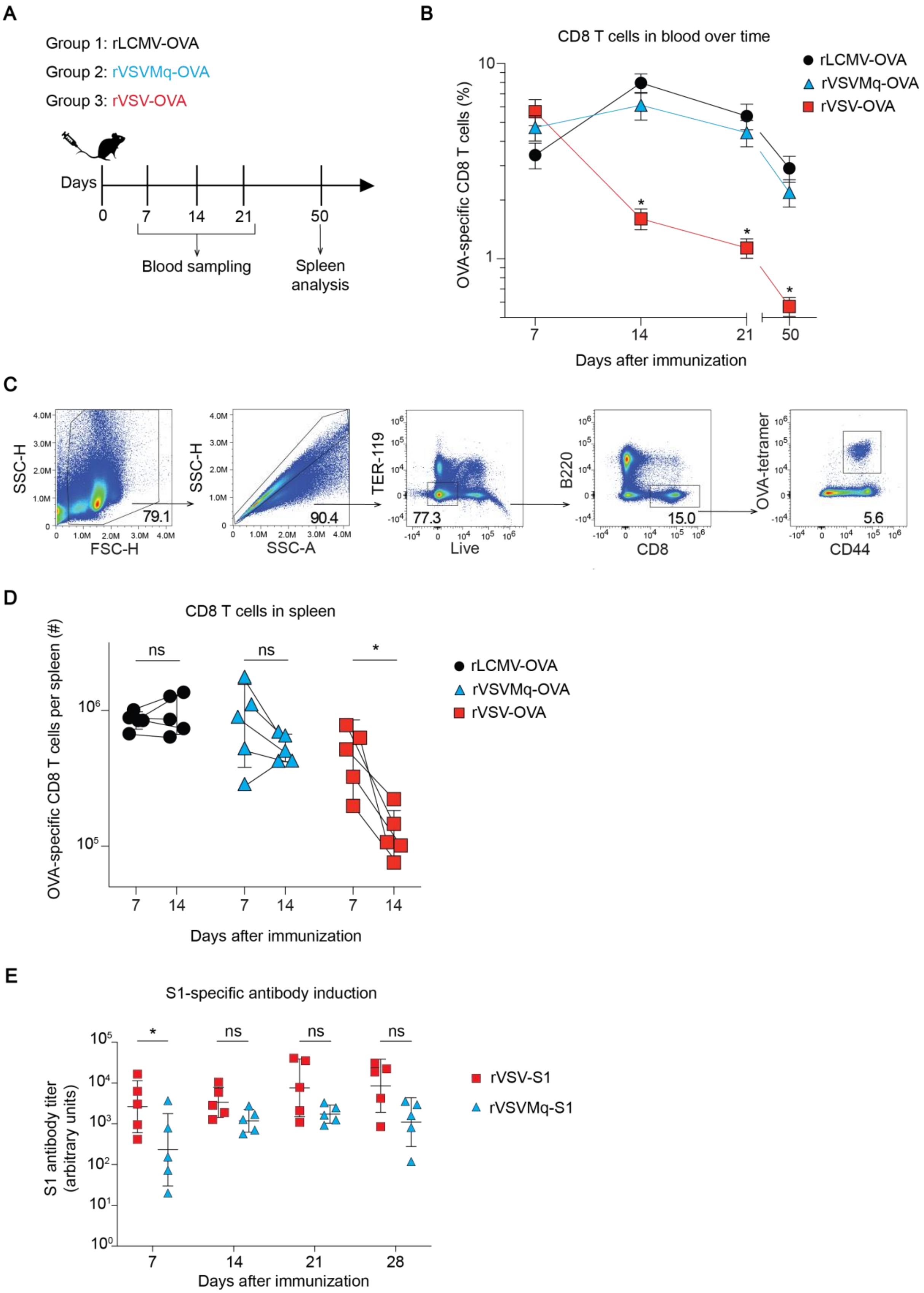

A
Group 1: rLCMV-OVA
Group 2: rVSVMq-OVA
Group 3: rVSV-OVA
Days
0
7
14
21
50
Blood sampling
Spleen analysis
B
CD8 T cells in blood over time
OVA-specific CD8 T cells (%)
rLCMV-OVA
rVSVMq-OVA
rVSV-OVA
Days after immunization
C
SSC-H
FSC-H
79.1
SSC-A
90.4
TER-119
Live
77.3
B220
CD8
15.0
OVA-tetramer
CD44
5.6
D
CD8 T cells in spleen
OVA-specific CD8 T cells per spleen (#)
ns
ns
*
rLCMV-OVA
rVSVMq-OVA
rVSV-OVA
Days after immunization
E
S1-specific antibody induction
S1 antibody titer (arbitrary units)
*
ns
ns
ns
rVSV-S1
rVSVMq-S1
Days after immunization

Figure S2. Kinetics of rVSV-, rVSV-Mq and rLCMV-induced CD8 T cell responses to OVA, and S1-specific antibody induction by rVSV- and rVSV-Mq. (A-C) We immunized mice i.v. with 1E+05 PFU LCMV-OVA or with 1E+06 PFU of either rVSVMq-OVA or rVSV-OVA on d0, and blood was sampled at several time points. In separate groups of mice, spleens were collected on d7 and d14, respectively. (B) Frequencies of OVA-tetramer-binding CD8 T cells in blood over time. (C) Total numbers of S1-tetramer-binding CD8 T cells in spleen. (E) Mice were immunized i.v. with rVSV-S1 or rVSVMq-S1, and blood was collected at different time points to determine S1-binding serum antibodies by ELISA. Symbols in (B) show the mean±SEM of 5 mice per group, symbols in (D,E) represent individual mice. One representative experiment of two similar ones is shown in (B, D). Statistical analyses were performed by two-way ANOVA with Bonferroni's post-test for multiple comparisons (B,D) or by one-way ANOVA followed by Tukey's post-test (C); **: $p < 0.01$; *: $p < 0.05$; ns.: $p > 0.05$.

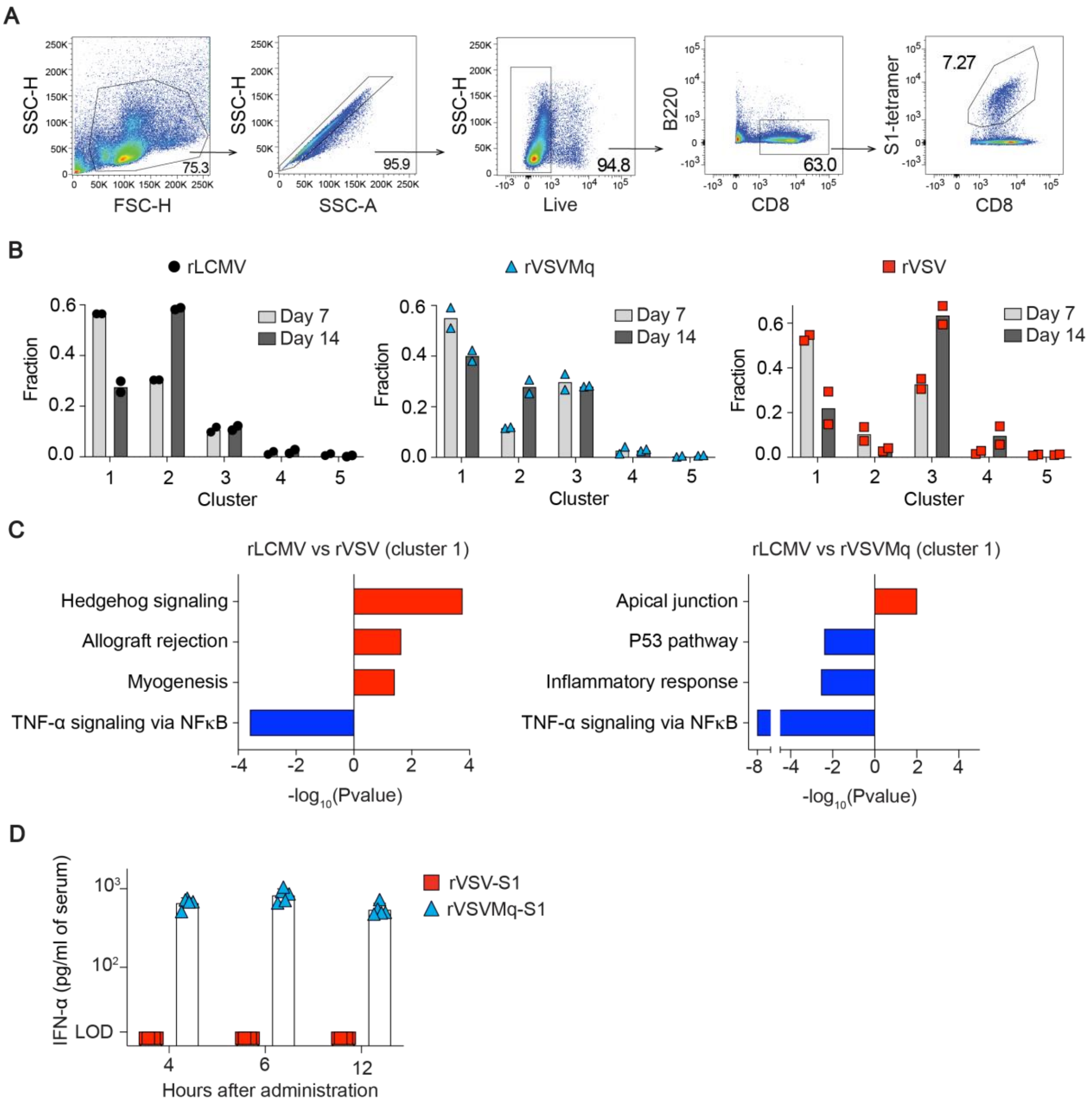

Figure S3. Gating strategy for FACS-sorting of epitope-specific CD8 T cells, vector-specific distribution of CD8 T cells in clusters, and kinetics of vector-induced IFN-α. (A) Gating strategy for FACS-sorting of tetramer-binding CD8 T cells to be processed for single cell RNA-sequencing. Prior to sorting, splenic single cell suspensions were enriched for CD8 T cells by magnetic-activated cell sorting. (B) Quantitative assessment of the distribution of rLCMV-S1-, rVSVMq-S1- and rVSV-S1-induced S1-epitope-specific CD8 T cells into clusters on d7 and on d14 of the experiment described in Fig. 3A-E. Symbols show individual mice (n=2 in each group), bars indicate the mean. Bars indicate for each cell cluster which fraction of the total S1-epitope-specific CD8 T cell population it represents. (C) Gene set enrichment analysis of cluster 1 cells, comparing rLCMV- to rVSV-induced CD8 T cells (left) and rLCMV- to rVSVMq-induced CD8 T cells (right). Gene sets with an FDR < 0.01 are displayed. (D) Time course

analysis of IFN-α levels in the serum of mice immunized with rVSV-S1 or rVSVMq-S1. Symbols show individual mice (n=4 in each group), bars indicate the group mean.

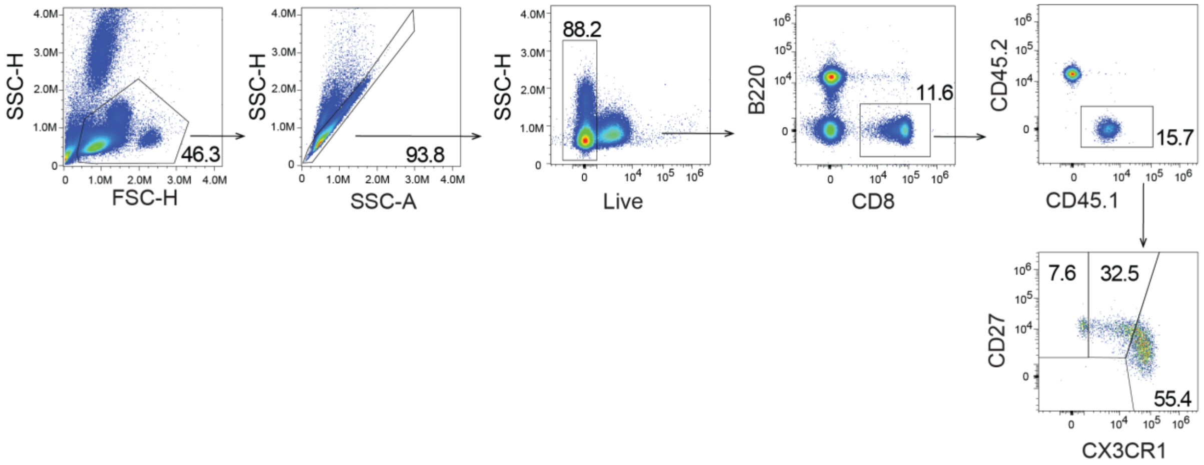

Figure S4. Gating strategy for the analysis of adoptively transferred OT-1 CD8 T cells.

Gating strategy for the analysis of adoptively transferred CD45.1^{+} OT-1 and OT-1x*Ifnar*$^{-/-}$ CD8 T cells in CD45.2-congenic recipients.

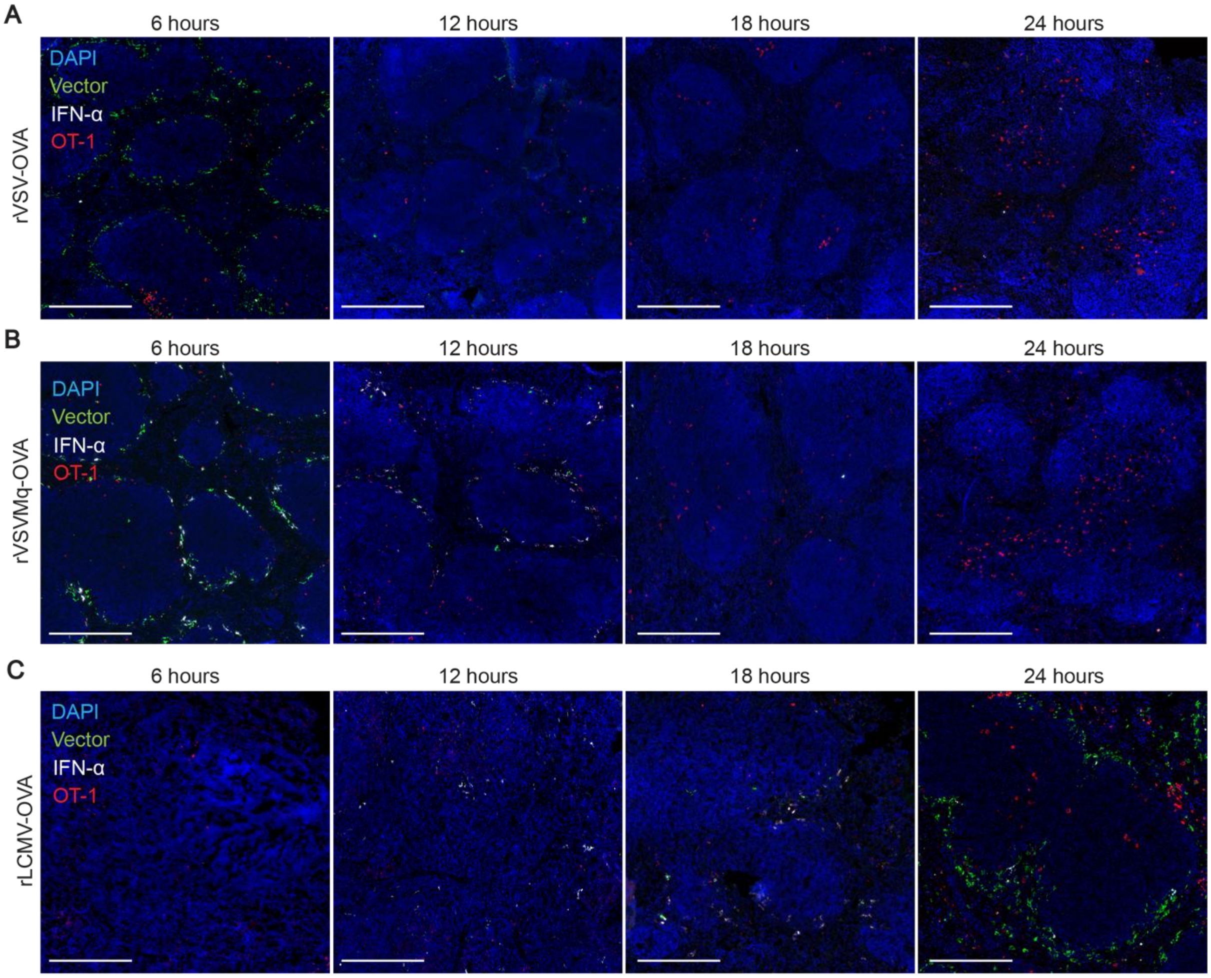

Figure S5. rVSVMq vaccination enables co-localization of cognate antigen and IFN-I near antigen-specific CD8 T cells. We transferred 2E+06 OT-1 cells to mice at -48h and immunized them with either rVSV-EGFP-OVA (A), rVSVMq-EGFP-OVA (B) or rLCMV-OVA (C) at 0h (same experiment as in Fig. 5). Spleens were collected at 6h, 12h, 18h and 24h. In (A,B) spleen sections were stained for cell nucleus (blue), IFN-α (white), CD45.1 (red; OT-1 cells) and GFP (vector infection; green). In (C) spleen sections were stained for cell nucleus (blue), IFN-α (white), CD45.1 (red; OT-1 cells) and LCMV nucleoprotein (vector infection; green). Magnification bars: 100 μm. Representative areas of spleen tissue from whole spleen sections of 4 mice per group from two combined experiments are shown.
